# Supplementary material for: Screening for genes that accelerate the epigenetic aging clock in humans reveals a role for the H3K36 methyltransferase NSD1
Source: Genome Biol. 2019 Aug 14;20:146. doi: 10.1186/s13059-019-1753-9 (PMC6693144; doi:10.1186/s13059-019-1753-9)
Supplement: Supplementary file 1 — Supplementary figures that complement the main manuscript. (PDF 2877 kb) [file 13059_2019_1753_MOESM1_ESM.pdf]

# ADDITIONAL FILE 1: SUPPLEMENTARY FIGURES

## Screening for genes that accelerate the epigenetic ageing clock in humans reveals a role for the H3K36 methyltransferase NSD1

Daniel E. Martin-Herranz[1,2], Erfan Aref-Eshghi[3,4], Marc Jan Bonder[1,5], Thomas M. Stubbs[2], Sanaa Choufani[6], Rosanna Weksberg[6], Oliver Stegle[1,5,7], Bekim Sadikovic[3,4], Wolf Reik[8,9,10,*], Janet M. Thornton[1,*]

[1]European Molecular Biology Laboratory, European Bioinformatics Institute, Wellcome Genome Campus, Hinxton, Cambridge, UK; [2]Chronomics Ltd, Cambridge, UK; [3]Department of Pathology and Laboratory Medicine, Western University, London, Canada; [4]Molecular Genetics Laboratory, Molecular Diagnostics Division, London Health Sciences Centre, London, Canada; [5]European Molecular Biology Laboratory, Genome Biology Unit, Heidelberg, Germany; [6]Genetics and Genome Biology Program, Research Institute, The Hospital for Sick Children, Toronto, Canada; [7]Division of Computational Genomics and Systems Genetics, German Cancer Research Center (DKFZ), Heidelberg, Germany; [8]Epigenetics Programme, The Babraham Institute, Cambridge, UK; [9]Centre for Trophoblast Research, University of Cambridge, Cambridge, UK; [10]Wellcome Sanger Institute, Hinxton, Cambridge, UK; *These authors jointly supervised this work

# Figure S1

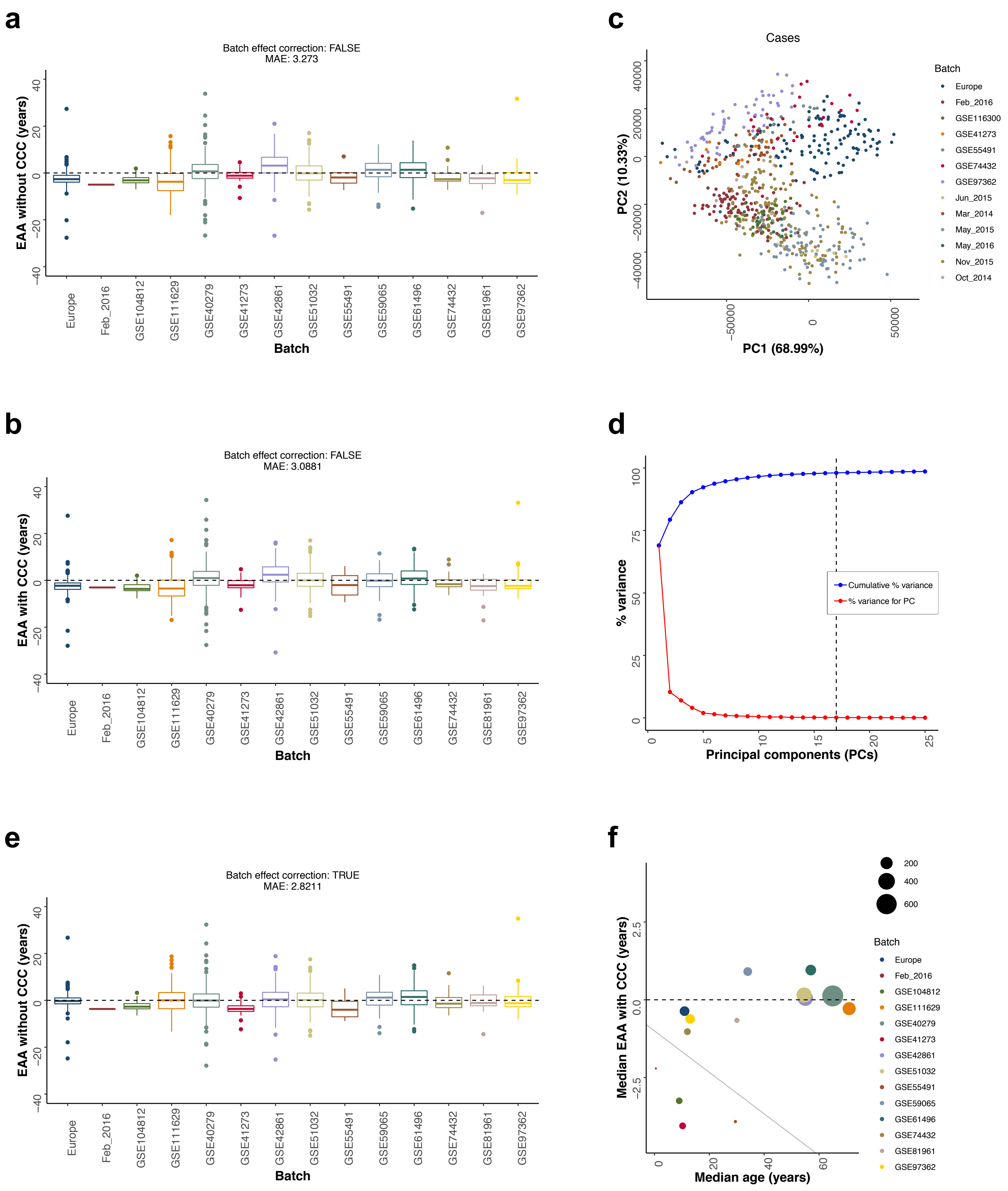

a
Batch effect correction: FALSE
MAE: 3.273
EAA without CCC (years)
Batch
Europe
Feb_2016
GSE104812
GSE111629
GSE40279
GSE41273
GSE42861
GSE51032
GSE55491
GSE59065
GSE61496
GSE74432
GSE81961
GSE97362
b
Batch effect correction: FALSE
MAE: 3.0881
EAA with CCC (years)
Batch
c
Cases
PC2 (10.33%)
PC1 (68.99%)
Batch
Europe
Feb_2016
GSE116300
GSE41273
GSE55491
GSE74432
GSE97362
Jun_2015
Mar_2014
May_2015
May_2016
Nov_2015
Oct_2014
d
% variance
Principal components (PCs)
Cumulative % variance
% variance for PC
e
Batch effect correction: TRUE
MAE: 2.8211
EAA without CCC (years)
Batch
f
Median EAA with CCC (years)
Median age (years)
200
400
600
Batch
Europe
Feb_2016
GSE104812
GSE111629
GSE40279
GSE41273
GSE42861
GSE51032
GSE55491
GSE59065
GSE61496
GSE74432
GSE81961
GSE97362

**Figure S1.** Complementary to Fig. 1. **a.** Distribution of the epigenetic age acceleration (EAA) without cell composition correction (CCC) for the different control batches, before applying batch effect correction. MAE: median absolute error. **b.** Distribution of the EAA with cell composition correction (CCC) for the different control batches, before applying batch effect correction. **c.** Scatterplot showing the values of the first two principal components (PCs) for the cases (developmental disorder samples) after performing PCA on the control probes of the 450K arrays. Each point corresponds to a different case sample and the colours represent the different batches. The different batches cluster together in the PCA space, showing that the control probes indeed capture technical variation. Please note that all the PCA calculations were done with more samples from cases and controls than those that were included in the final screening since it was performed before the filtering step (see Methods). **d.** Plot showing the percentages of technical variance explained by the different PCs from the control probes. The dashed line represents the optimal number of PCs (17) that was finally used. **e.** Distribution of the EAA without cell composition correction (CCC) for the different control batches, after applying batch effect correction. **f.** After batch effect correction, deviations from a median EAA of zero (dotted black line) in some of the control batches can be explained by other causes. The grey line separates in the lower left corner those weird batches (Feb_2016, GSE104812, GSE41273, GSE55491), which have a small sample size and/or a low median age.

# Figure S2

**a**

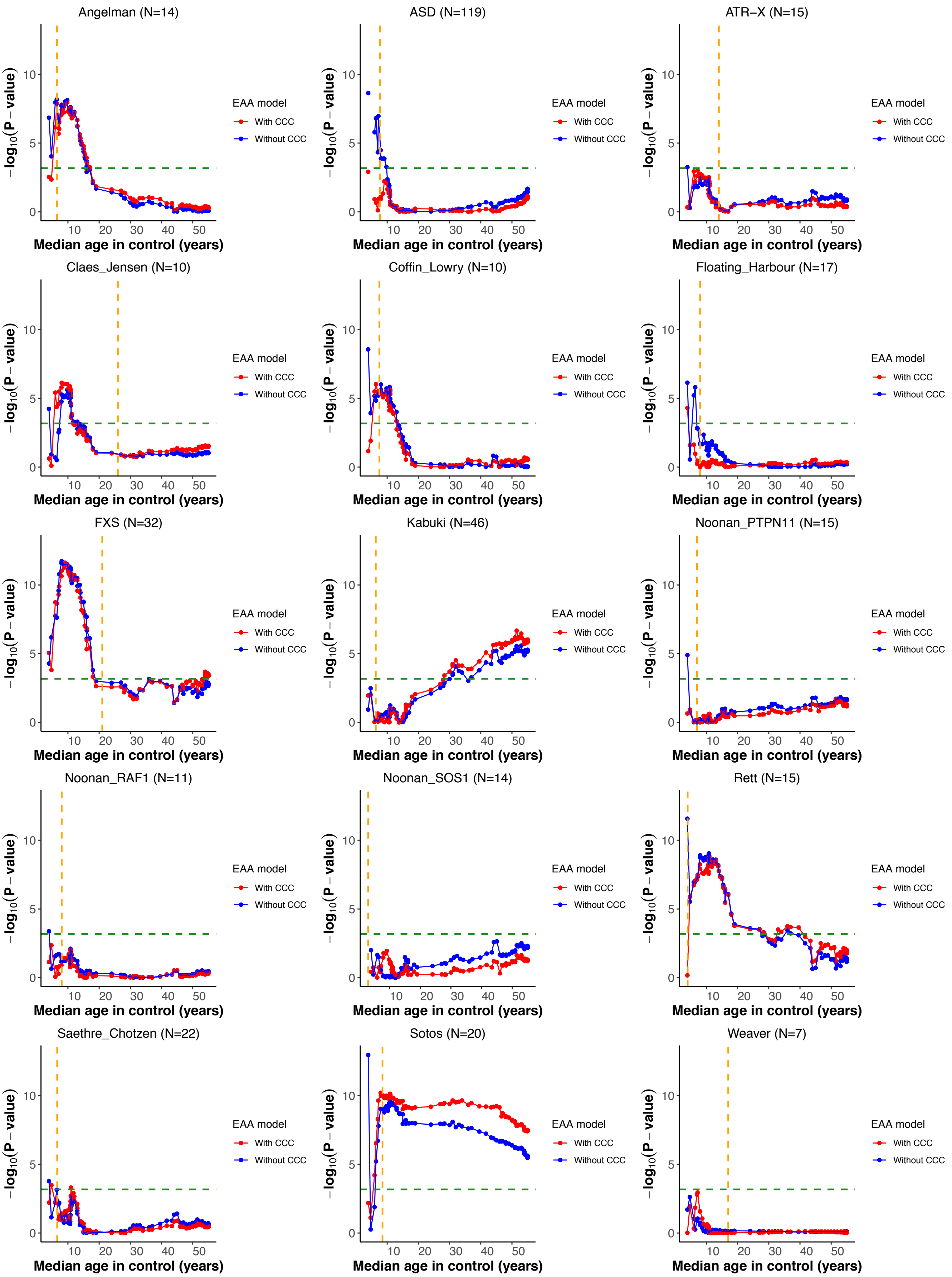

Angelman (N=14)
ASD (N=119)
ATR−X (N=15)
Claes_Jensen (N=10)
Coffin_Lowry (N=10)
Floating_Harbour (N=17)
FXS (N=32)
Kabuki (N=46)
Noonan_PTPN11 (N=15)
Noonan_RAF1 (N=11)
Noonan_SOS1 (N=14)
Rett (N=15)
Saethre_Chotzen (N=22)
Sotos (N=20)
Weaver (N=7)
$-\log_{10}(P-\text{value})$
Median age in control (years)
EAA model
With CCC
Without CCC

**b**

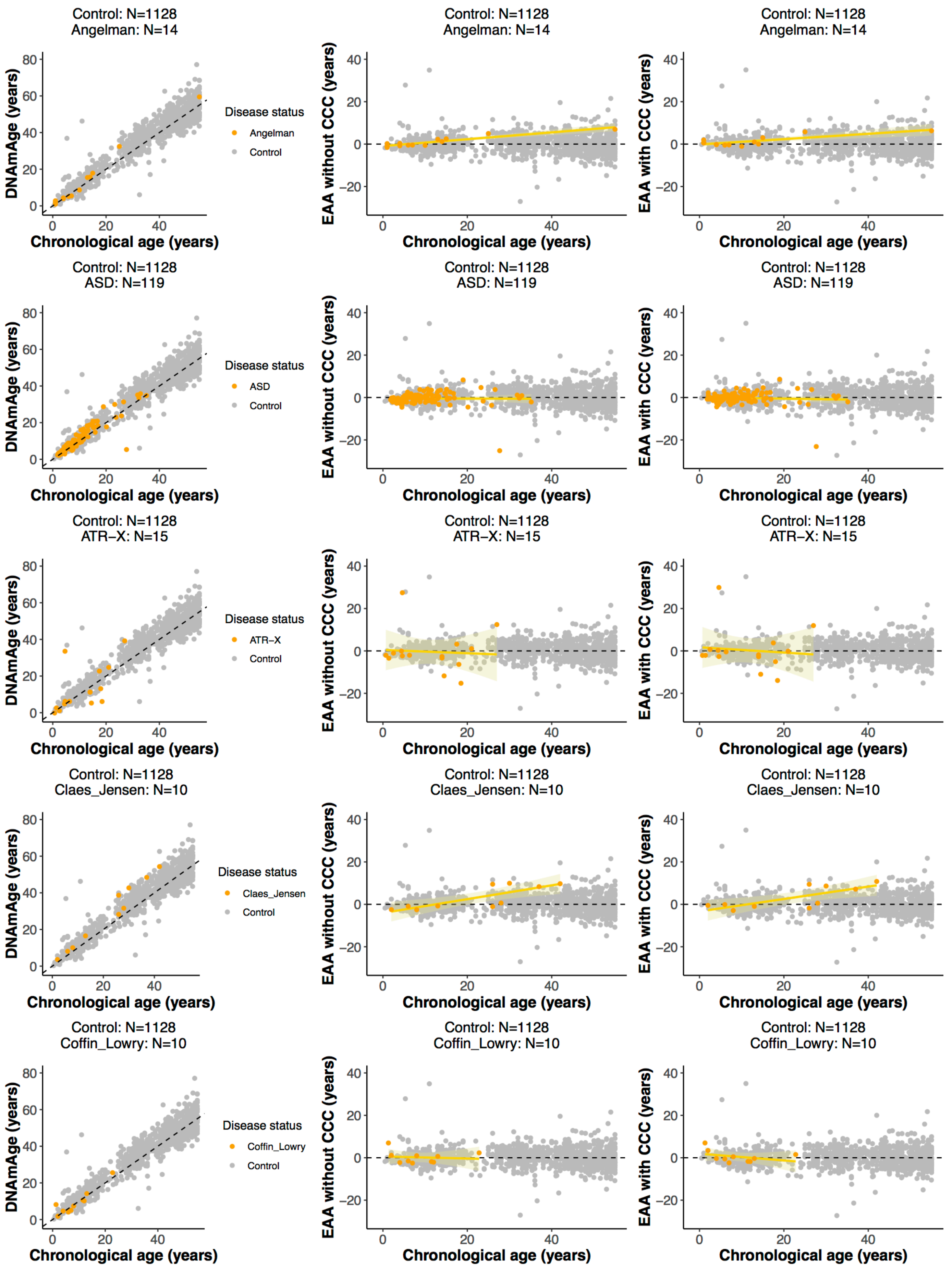
Control: N=1128
Angelman: N=14
DNAmAge (years)
Chronological age (years)
Disease status
Angelman
Control
EAA without CCC (years)
EAA with CCC (years)
Control: N=1128
ASD: N=119
ASD
Control: N=1128
ATR−X: N=15
ATR−X
Control: N=1128
Claes_Jensen: N=10
Claes_Jensen
Control: N=1128
Coffin_Lowry: N=10
Coffin_Lowry

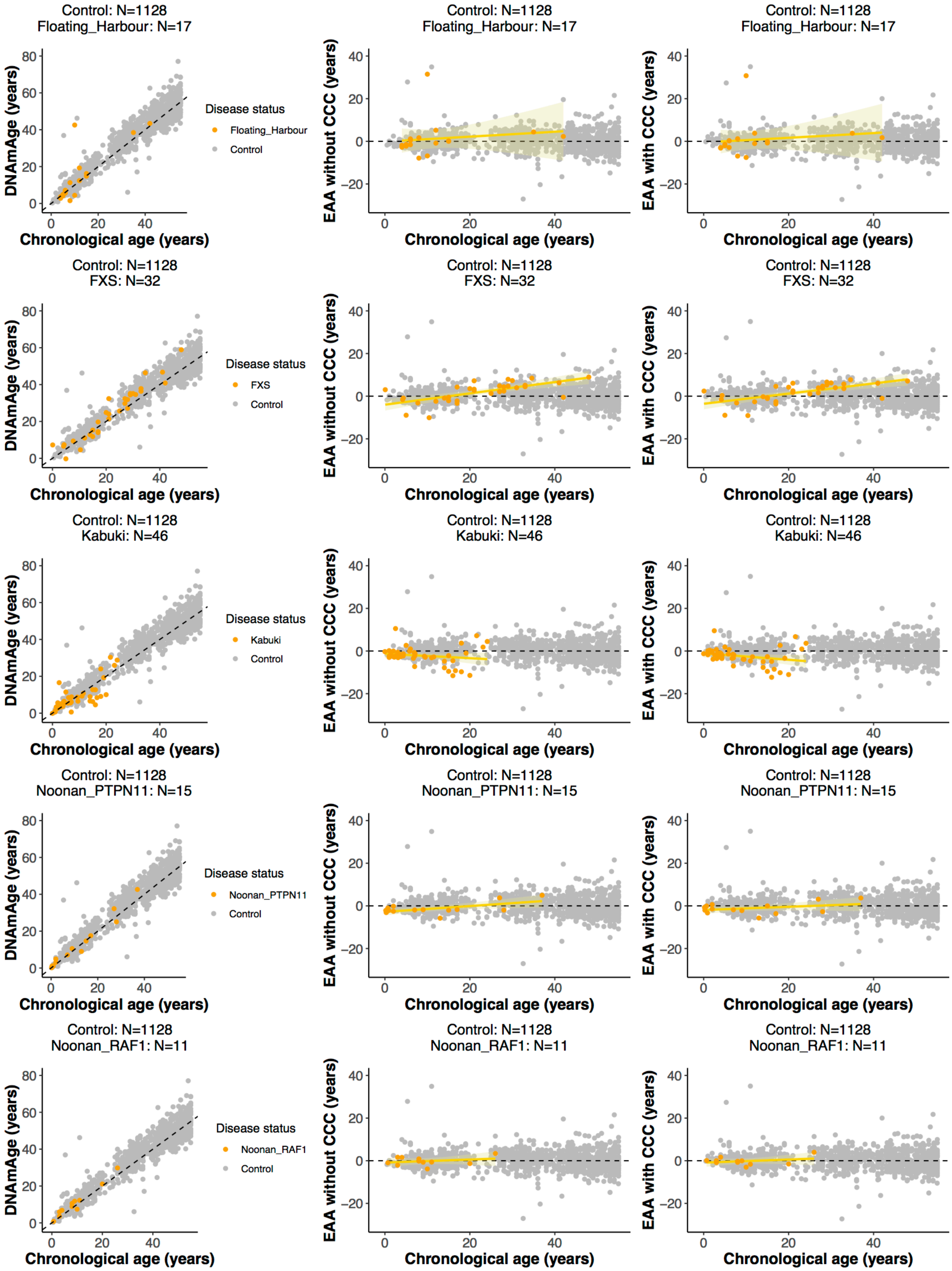
Control: N=1128
Floating_Harbour: N=17
DNAmAge (years)
Chronological age (years)
Disease status
Floating_Harbour
Control
EAA without CCC (years)
EAA with CCC (years)
Control: N=1128
FXS: N=32
Disease status
FXS
Control
Control: N=1128
Kabuki: N=46
Disease status
Kabuki
Control
Control: N=1128
Noonan_PTPN11: N=15
Disease status
Noonan_PTPN11
Control
Control: N=1128
Noonan_RAF1: N=11
Disease status
Noonan_RAF1
Control

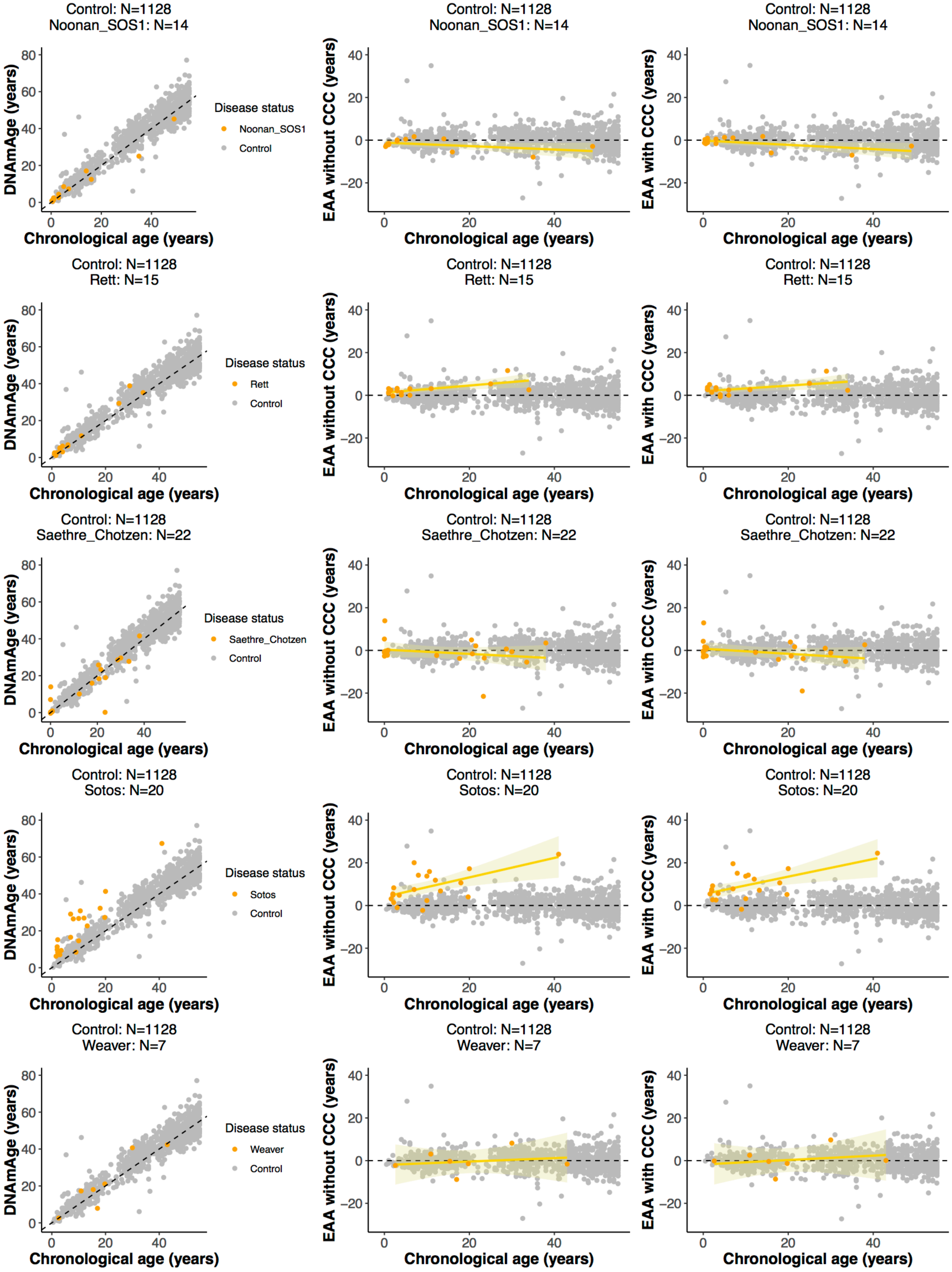
Control: N=1128
Noonan_SOS1: N=14
DNAmAge (years)
Chronological age (years)
Disease status
Noonan_SOS1
Control
EAA without CCC (years)
EAA with CCC (years)
Control: N=1128
Rett: N=15
Disease status
Rett
Control
Control: N=1128
Saethre_Chotzen: N=22
Disease status
Saethre_Chotzen
Control
Control: N=1128
Sotos: N=20
Disease status
Sotos
Control
Control: N=1128
Weaver: N=7
Disease status
Weaver
Control

**c**

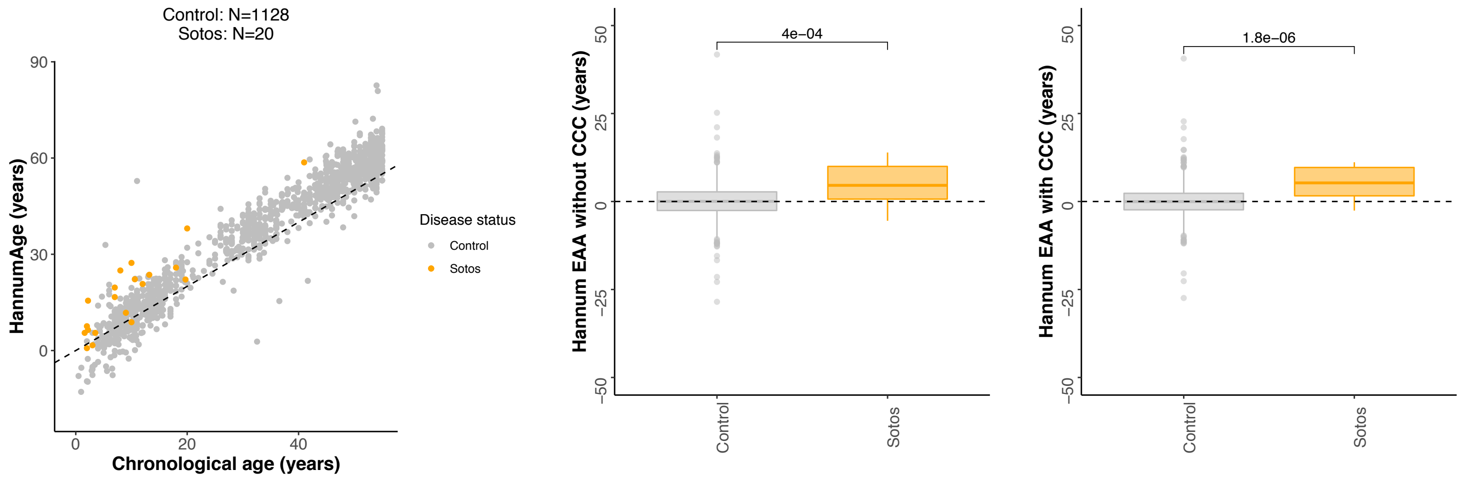
Control: N=1128
Sotos: N=20
HannumAge (years)
Chronological age (years)
Disease status
Control
Sotos
Hannum EAA without CCC (years)
4e−04
Hannum EAA with CCC (years)
1.8e−06
Control
Sotos

**d**

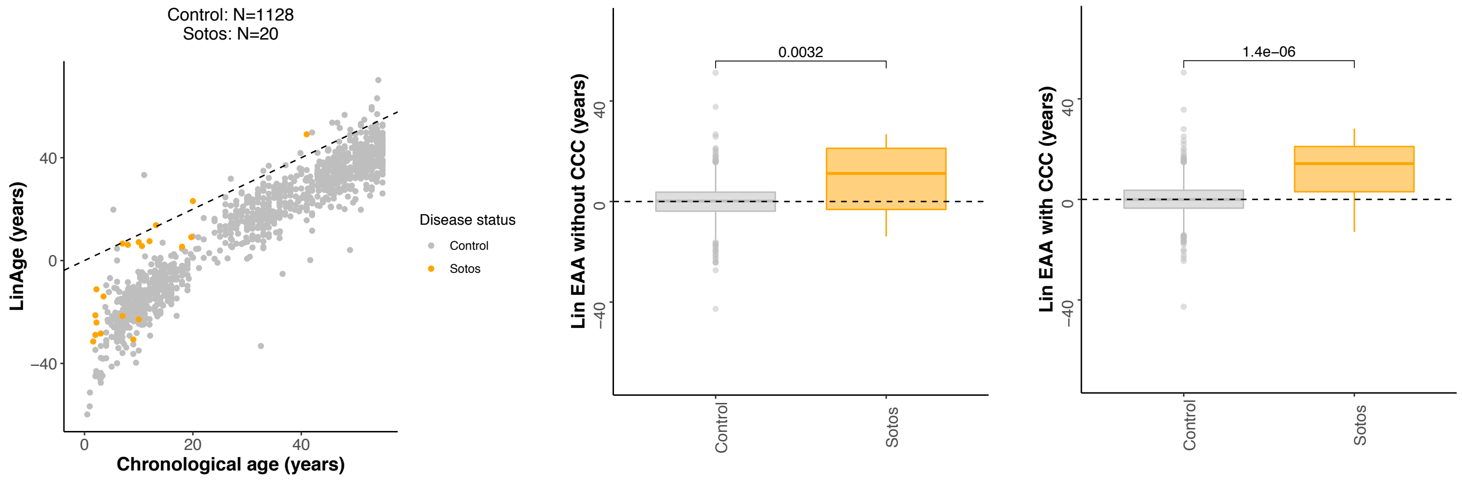
Control: N=1128
Sotos: N=20
LinAge (years)
Chronological age (years)
Disease status
Control
Sotos
Lin EAA without CCC (years)
0.0032
Lin EAA with CCC (years)
1.4e−06
Control
Sotos

**e**

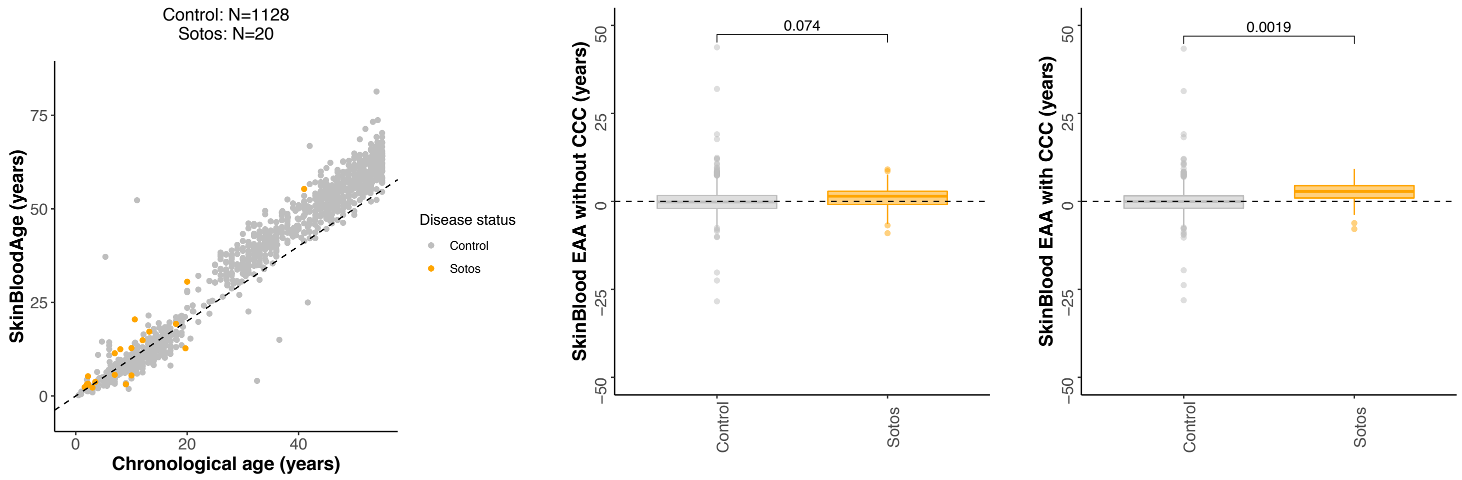
Control: N=1128
Sotos: N=20
SkinBloodAge (years)
Chronological age (years)
Disease status
Control
Sotos
SkinBlood EAA without CCC (years)
0.074
SkinBlood EAA with CCC (years)
0.0019
Control
Sotos

**Figure S2.** Complementary to Fig. 2. **a.** Effect of changing the median age of the controls when performing the screening for epigenetic age acceleration (EAA) in the different developmental disorders. The dashed green line displays the significance level of $\alpha = 0.01$ after Bonferroni correction. The dashed orange line displays the median age for the samples in the developmental disorder considered. In blue: EAA model without cell composition correction (CCC). In red: EAA model with CCC. **b.** Left panel: scatterplot showing the relation between epigenetic age (*DNAmAge*) according to Horvath's model and chronological age of the samples for a given developmental disorder (orange) and control (grey). Each sample is represented by one point. The black dashed line represents the diagonal to aid visualisation. Middle and right panels: scatterplots showing the relation between the epigenetic age acceleration (EAA) (without and with CCC respectively) and chronological age of the samples for a given developmental disorder (orange) and control (grey). Each sample is represented by one point. The yellow line represents the linear model *EAA ~ Age*, with the standard error shown in the light yellow shade. **c.** Left panel: scatterplot showing the relation between epigenetic age (*HannumAge*) according to Hannum's model and chronological age of the samples for Sotos (orange) and control (grey). Each sample is represented by one point. The black dashed line represents the diagonal to aid visualisation. Middle and right panels: boxplots showing the comparisons of the EAA distributions (according to Hannum's clock) for Sotos and control samples (without and with CCC respectively). The p-values (two-sided Wilcoxon's test, before multiple testing correction) are shown above the boxplots. **d.** As in c., but using Lin's epigenetic clock. **e.** As in c., but using the skin-blood epigenetic clock.

# Figure S3

a

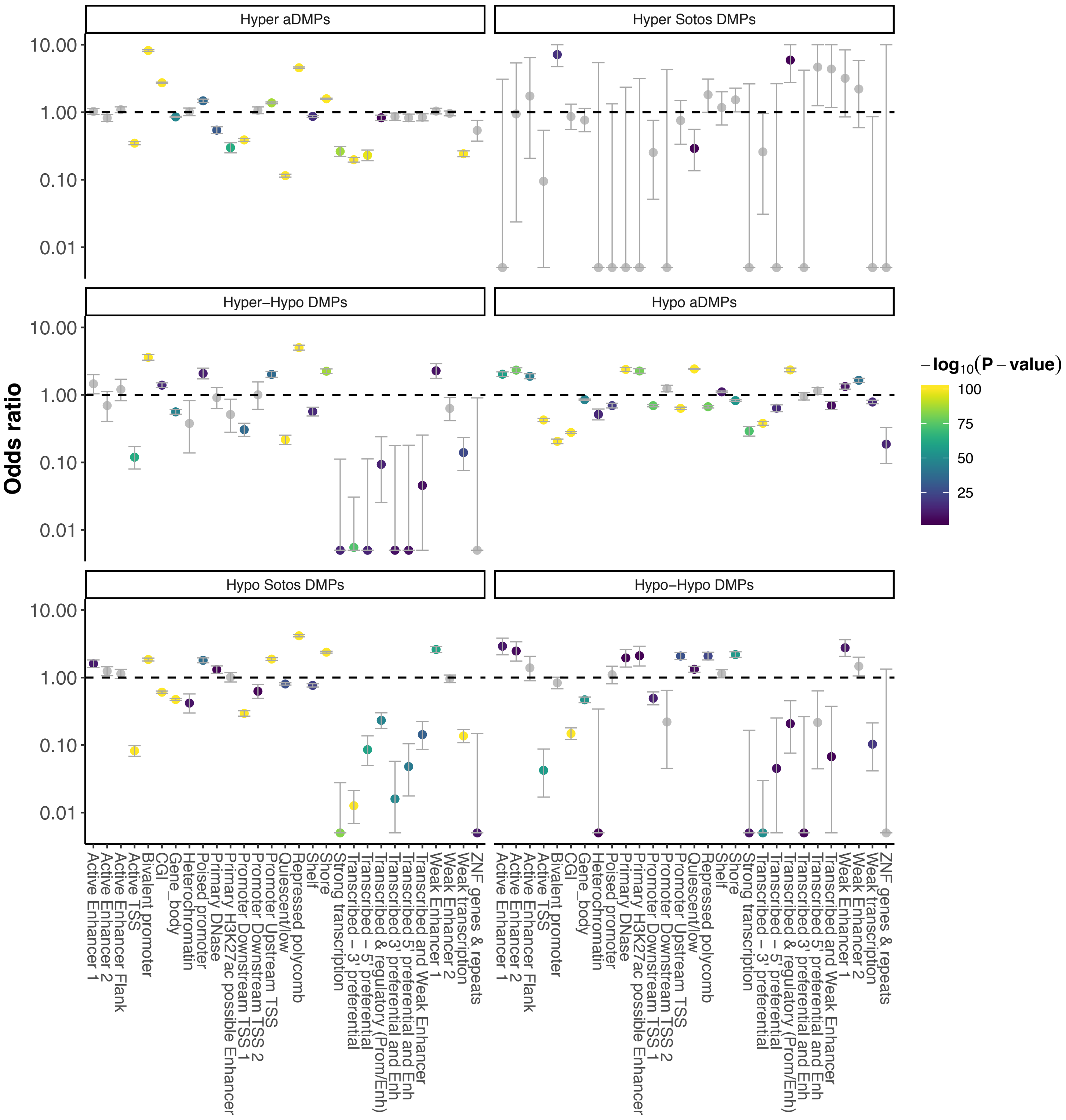
Hyper aDMPs
Hyper Sotos DMPs
Hyper−Hypo DMPs
Hypo aDMPs
Hypo Sotos DMPs
Hypo−Hypo DMPs
Odds ratio
10.00
1.00
0.10
0.01
$-\log_{10}(P-value)$
100
75
50
25
Active Enhancer 1
Active Enhancer 2
Active Enhancer Flank
Active TSS
Bivalent promoter
CGI
Gene_body
Heterochromatin
Poised promoter
Primary DNase
Primary H3K27ac possible Enhancer
Promoter Downstream TSS 1
Promoter Downstream TSS 2
Promoter Upstream TSS
Quiescent/low
Repressed polycomb
Shelf
Shore
Strong transcription
Transcribed − 3' preferential
Transcribed − 5' preferential
Transcribed & regulatory (Prom/Enh)
Transcribed 3' preferential and Enh
Transcribed 5' preferential and Enh
Transcribed and Weak Enhancer
Weak Enhancer 1
Weak Enhancer 2
Weak transcription
ZNF genes & repeats

**b**

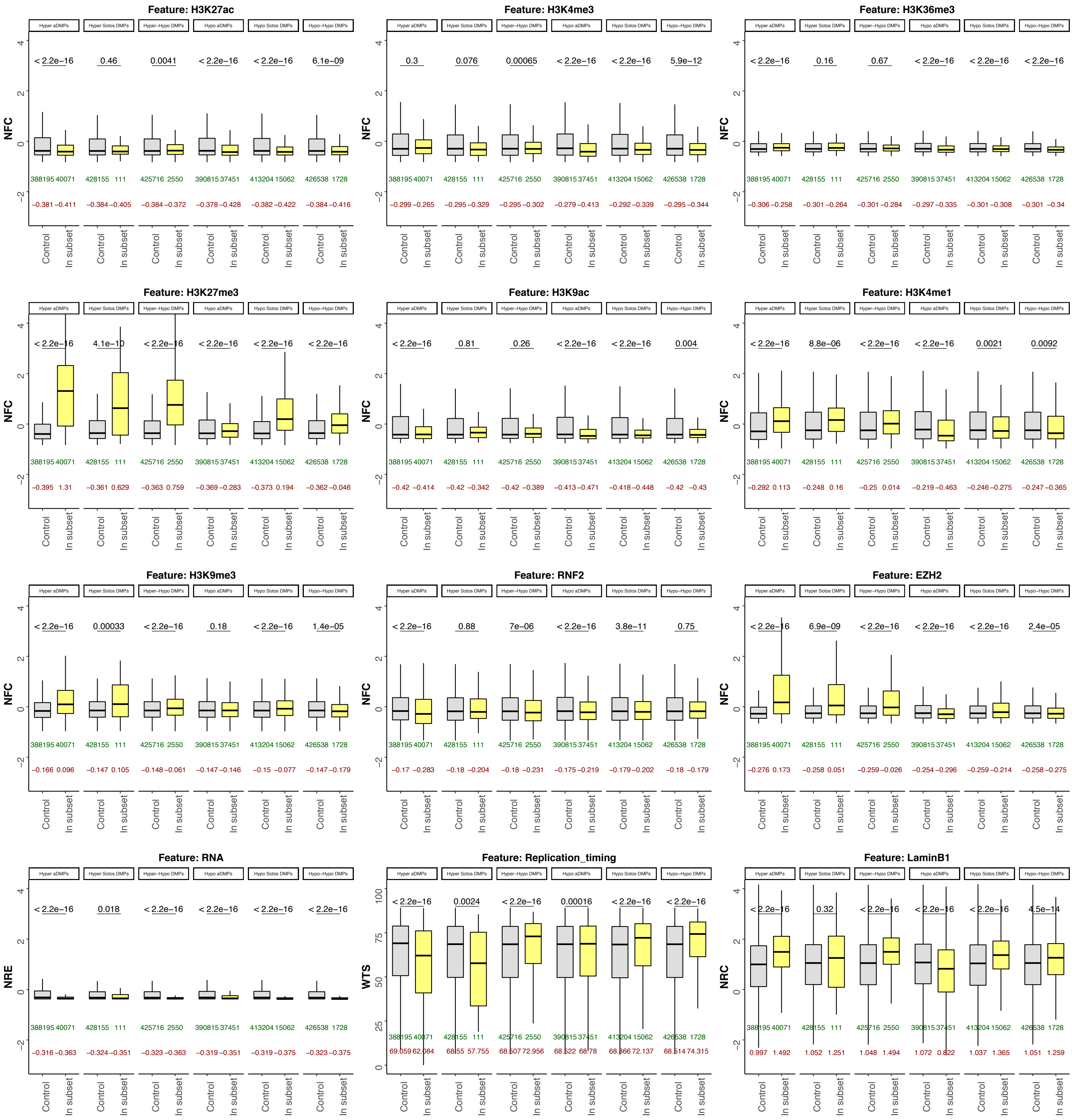
Feature: H3K27ac
Hyper aDMPs
Hyper Sotos DMPs
Hyper–Hypo DMPs
Hypo aDMPs
Hypo Sotos DMPs
Hypo–Hypo DMPs
NFC
< 2.2e–16
0.46
0.0041
< 2.2e–16
< 2.2e–16
6.1e–09
388195 40071
428155 111
425716 2550
390815 37451
413204 15062
426538 1728
−0.381 −0.411
−0.384 −0.405
−0.384 −0.372
−0.378 −0.428
−0.382 −0.422
−0.384 −0.416
Control
In subset
Feature: H3K4me3
0.3
0.076
0.00065
< 2.2e–16
< 2.2e–16
5.9e–12
−0.299 −0.265
−0.295 −0.329
−0.295 −0.302
−0.279 −0.413
−0.292 −0.339
−0.295 −0.344
Feature: H3K36me3
< 2.2e–16
0.16
0.67
< 2.2e–16
< 2.2e–16
< 2.2e–16
−0.306 −0.258
−0.301 −0.264
−0.301 −0.284
−0.297 −0.335
−0.301 −0.308
−0.301 −0.34
Feature: H3K27me3
< 2.2e–16
4.1e–10
< 2.2e–16
< 2.2e–16
< 2.2e–16
< 2.2e–16
−0.395 1.31
−0.361 0.629
−0.363 0.759
−0.369 −0.283
−0.373 0.194
−0.362 −0.046
Feature: H3K9ac
< 2.2e–16
0.81
0.26
< 2.2e–16
< 2.2e–16
0.004
−0.42 −0.414
−0.42 −0.342
−0.42 −0.389
−0.413 −0.471
−0.418 −0.448
−0.42 −0.43
Feature: H3K4me1
< 2.2e–16
8.8e–06
< 2.2e–16
< 2.2e–16
0.0021
0.0092
−0.292 0.113
−0.248 0.16
−0.25 0.014
−0.219 −0.463
−0.246 −0.275
−0.247 −0.365
Feature: H3K9me3
< 2.2e–16
0.00033
< 2.2e–16
0.18
< 2.2e–16
1.4e–05
−0.166 0.096
−0.147 0.105
−0.148 −0.061
−0.147 −0.146
−0.15 −0.077
−0.147 −0.179
Feature: RNF2
< 2.2e–16
0.88
7e–06
< 2.2e–16
3.8e–11
0.75
−0.17 −0.283
−0.18 −0.204
−0.18 −0.231
−0.175 −0.219
−0.179 −0.202
−0.18 −0.179
Feature: EZH2
< 2.2e–16
6.9e–09
< 2.2e–16
< 2.2e–16
< 2.2e–16
2.4e–05
−0.276 0.173
−0.258 0.051
−0.259 −0.026
−0.254 −0.296
−0.259 −0.214
−0.258 −0.275
Feature: RNA
NRE
< 2.2e–16
0.018
< 2.2e–16
< 2.2e–16
< 2.2e–16
< 2.2e–16
−0.316 −0.363
−0.324 −0.351
−0.323 −0.363
−0.319 −0.351
−0.319 −0.375
−0.323 −0.375
Feature: Replication_timing
WTS
< 2.2e–16
0.0024
< 2.2e–16
0.00016
< 2.2e–16
< 2.2e–16
69.059 62.084
68.55 57.755
68.607 72.956
68.522 68.78
68.866 72.137
68.614 74.315
Feature: LaminB1
NRC
< 2.2e–16
0.32
< 2.2e–16
< 2.2e–16
< 2.2e–16
4.5e–14
0.997 1.492
1.052 1.251
1.048 1.494
1.072 0.822
1.037 1.365
1.051 1.259

c

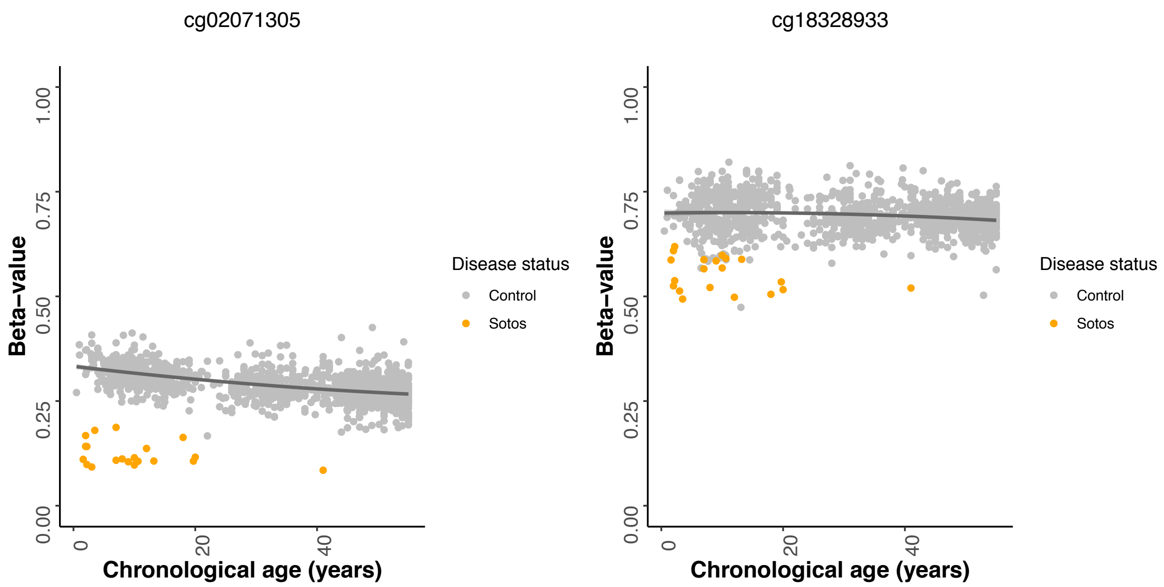

cg02071305
cg18328933
Beta−value
0.00
0.25
0.50
0.75
1.00
0
20
40
Chronological age (years)
Disease status
Control
Sotos


d

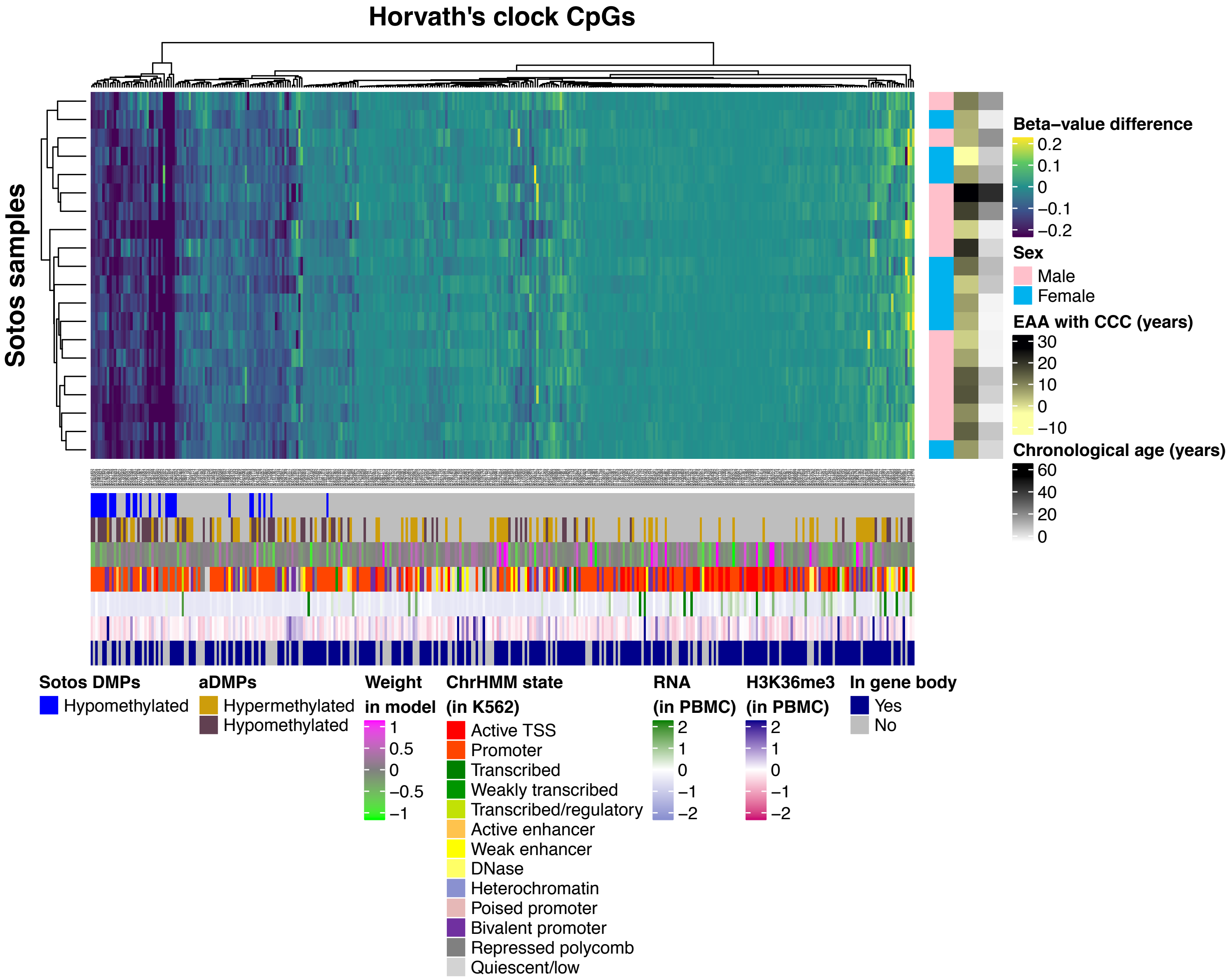

Horvath's clock CpGs
Sotos samples
Beta−value difference
0.2
0.1
0
−0.1
−0.2
Sex
Male
Female
EAA with CCC (years)
30
20
10
0
−10
Chronological age (years)
60
40
20
0
Sotos DMPs
Hypomethylated
aDMPs
Hypermethylated
Hypomethylated
Weight in model
1
0.5
0
−0.5
−1
ChrHMM state (in K562)
Active TSS
Promoter
Transcribed
Weakly transcribed
Transcribed/regulatory
Active enhancer
Weak enhancer
DNase
Heterochromatin
Poised promoter
Bivalent promoter
Repressed polycomb
Quiescent/low
RNA (in PBMC)
2
1
0
−1
−2
H3K36me3 (in PBMC)
2
1
0
−1
−2
In gene body
Yes
No

e

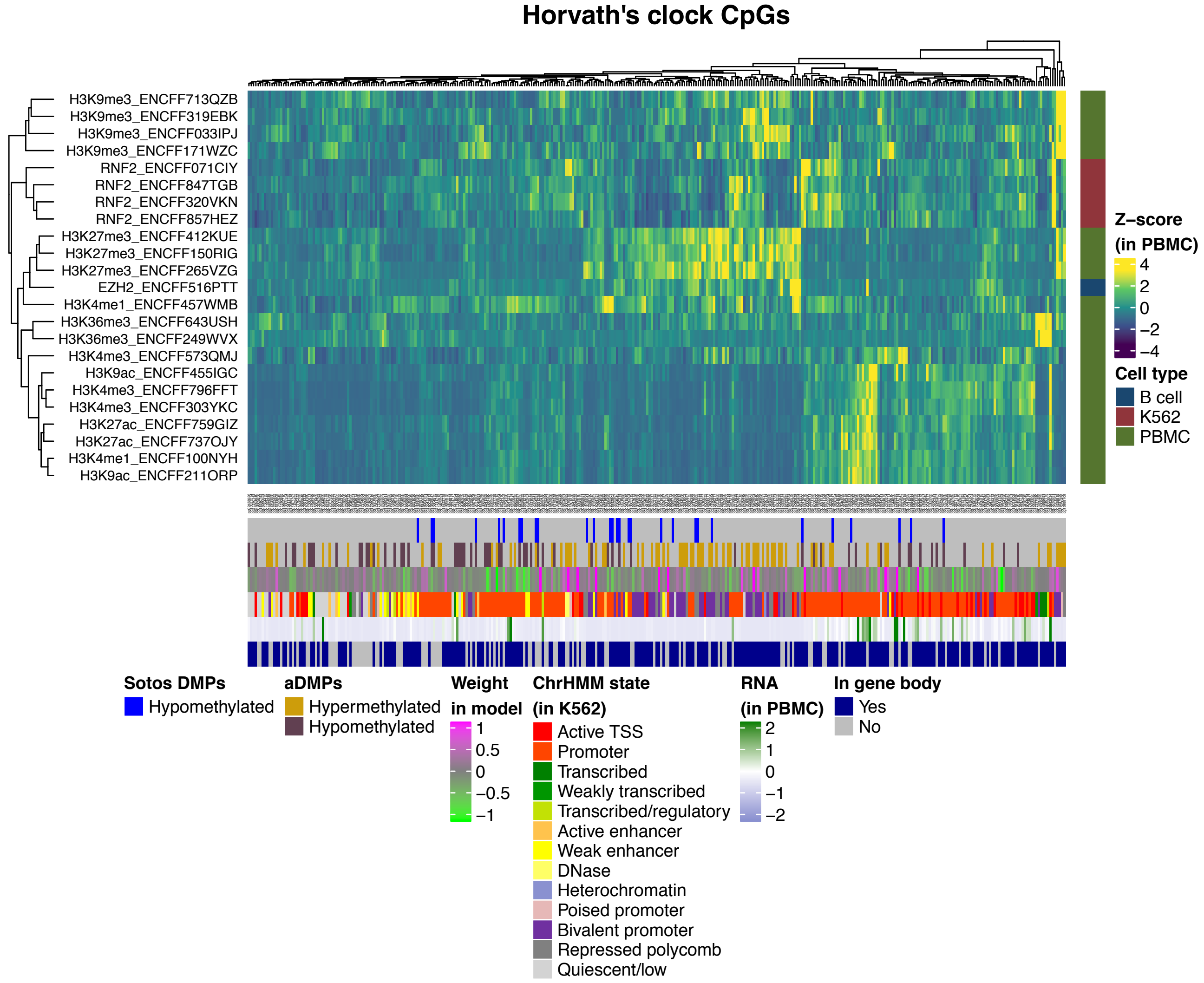

Horvath's clock CpGs
Features
H3K9me3_ENCFF713QZB
H3K9me3_ENCFF319EBK
H3K9me3_ENCFF033IPJ
H3K9me3_ENCFF171WZC
RNF2_ENCFF071CIY
RNF2_ENCFF847TGB
RNF2_ENCFF320VKN
RNF2_ENCFF857HEZ
H3K27me3_ENCFF412KUE
H3K27me3_ENCFF150RIG
H3K27me3_ENCFF265VZG
EZH2_ENCFF516PTT
H3K4me1_ENCFF457WMB
H3K36me3_ENCFF643USH
H3K36me3_ENCFF249WVX
H3K4me3_ENCFF573QMJ
H3K9ac_ENCFF455IGC
H3K4me3_ENCFF796FFT
H3K4me3_ENCFF303YKC
H3K27ac_ENCFF759GIZ
H3K27ac_ENCFF737OJY
H3K4me1_ENCFF100NYH
H3K9ac_ENCFF211ORP
Z–score (in PBMC)
4
2
0
−2
−4
Cell type
B cell
K562
PBMC
Sotos DMPs
Hypomethylated
aDMPs
Hypermethylated
Hypomethylated
Weight in model
1
0.5
0
−0.5
−1
ChrHMM state (in K562)
Active TSS
Promoter
Transcribed
Weakly transcribed
Transcribed/regulatory
Active enhancer
Weak enhancer
DNase
Heterochromatin
Poised promoter
Bivalent promoter
Repressed polycomb
Quiescent/low
RNA (in PBMC)
2
1
0
−1
−2
In gene body
Yes
No


f

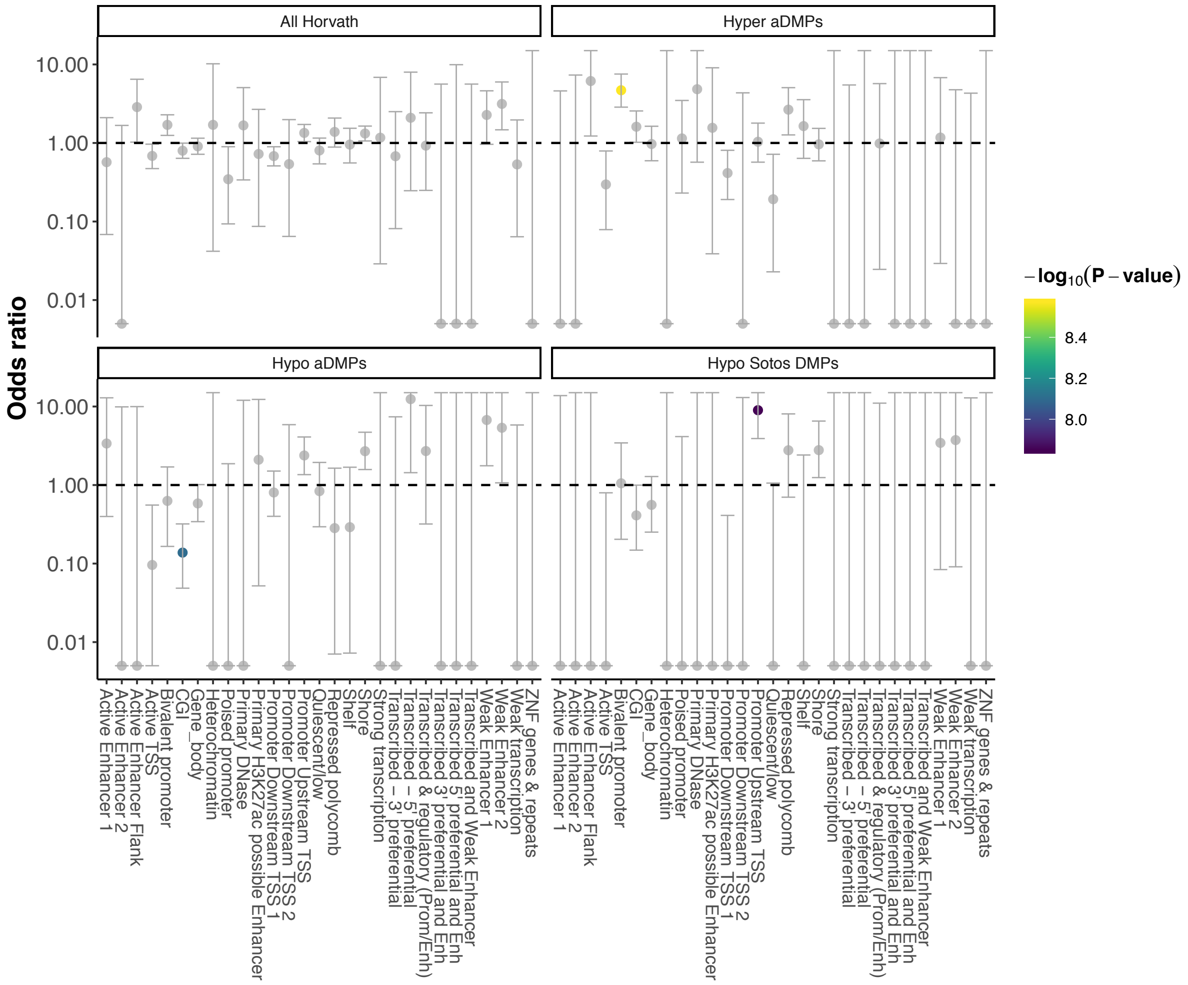

All Horvath
Hyper aDMPs
Hypo aDMPs
Hypo Sotos DMPs
Odds ratio
10.00
1.00
0.10
0.01
−log10(P − value)
8.4
8.2
8.0
Active Enhancer 1
Active Enhancer 2
Active Enhancer Flank
Active TSS
Bivalent promoter
CGI
Gene_body
Heterochromatin
Poised promoter
Primary DNase
Primary H3K27ac possible Enhancer
Promoter Downstream TSS 1
Promoter Downstream TSS 2
Promoter Upstream TSS
Quiescent/low
Repressed polycomb
Shelf
Shore
Strong transcription
Transcribed − 3' preferential
Transcribed − 5' preferential
Transcribed & regulatory (Prom/Enh)
Transcribed 3' preferential and Enh
Transcribed 5' preferential and Enh
Transcribed and Weak Enhancer
Weak Enhancer 1
Weak Enhancer 2
Weak transcription
ZNF genes & repeats

**g**

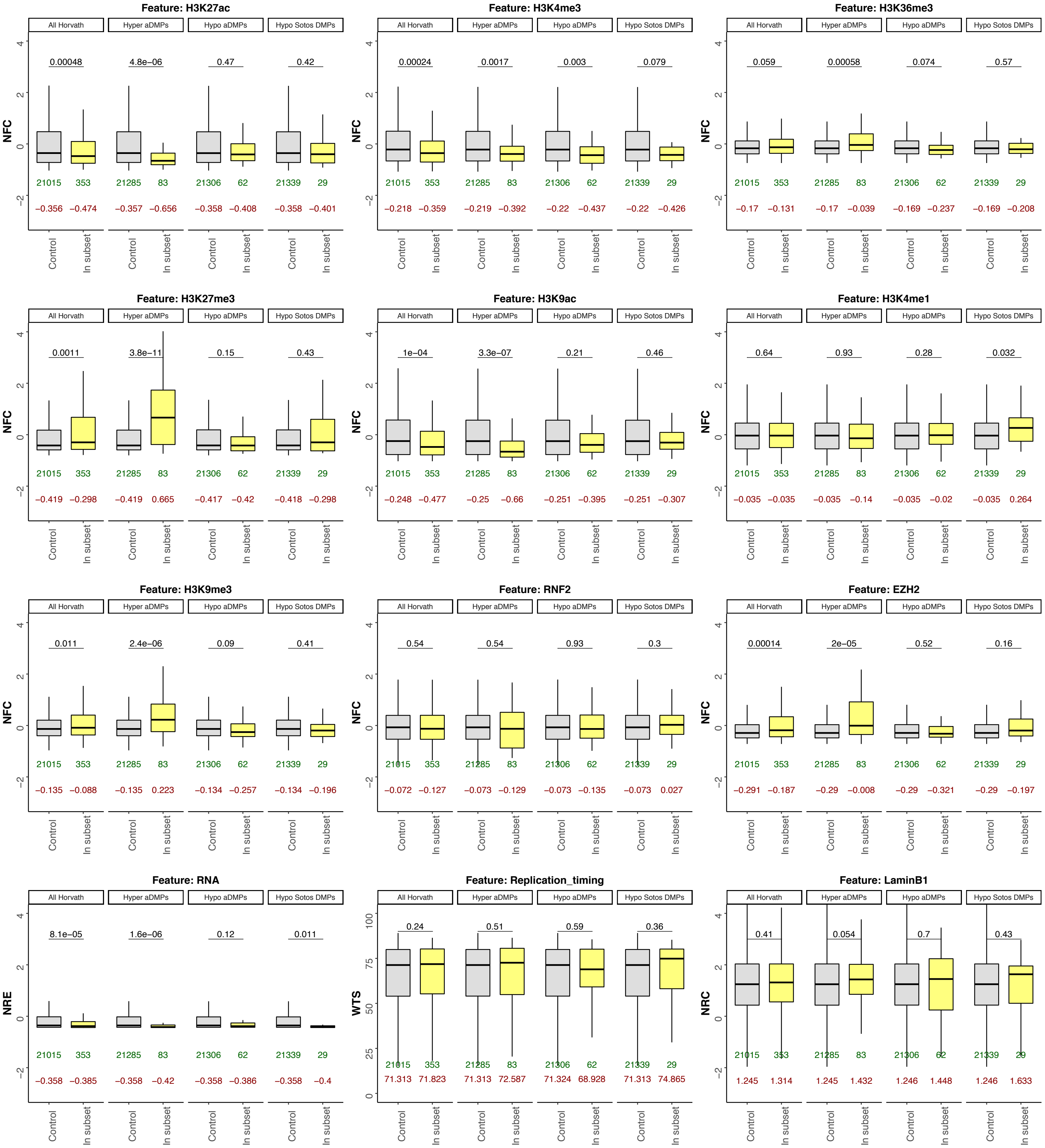
Feature: H3K27ac
All Horvath
Hyper aDMPs
Hypo aDMPs
Hypo Sotos DMPs
0.00048
4.8e−06
0.47
0.42
NFC
21015 353 21285 83 21306 62 21339 29
−0.356 −0.474 −0.357 −0.656 −0.358 −0.408 −0.358 −0.401
Control
In subset
Feature: H3K4me3
0.00024
0.0017
0.003
0.079
21015 353 21285 83 21306 62 21339 29
−0.218 −0.359 −0.219 −0.392 −0.22 −0.437 −0.22 −0.426
Feature: H3K36me3
0.059
0.00058
0.074
0.57
21015 353 21285 83 21306 62 21339 29
−0.17 −0.131 −0.17 −0.039 −0.169 −0.237 −0.169 −0.208
Feature: H3K27me3
0.0011
3.8e−11
0.15
0.43
21015 353 21285 83 21306 62 21339 29
−0.419 −0.298 −0.419 0.665 −0.417 −0.42 −0.418 −0.298
Feature: H3K9ac
1e−04
3.3e−07
0.21
0.46
21015 353 21285 83 21306 62 21339 29
−0.248 −0.477 −0.25 −0.66 −0.251 −0.395 −0.251 −0.307
Feature: H3K4me1
0.64
0.93
0.28
0.032
21015 353 21285 83 21306 62 21339 29
−0.035 −0.035 −0.035 −0.14 −0.035 −0.02 −0.035 0.264
Feature: H3K9me3
0.011
2.4e−06
0.09
0.41
21015 353 21285 83 21306 62 21339 29
−0.135 −0.088 −0.135 0.223 −0.134 −0.257 −0.134 −0.196
Feature: RNF2
0.54
0.54
0.93
0.3
21015 353 21285 83 21306 62 21339 29
−0.072 −0.127 −0.073 −0.129 −0.073 −0.135 −0.073 0.027
Feature: EZH2
0.00014
2e−05
0.52
0.16
21015 353 21285 83 21306 62 21339 29
−0.291 −0.187 −0.29 −0.008 −0.29 −0.321 −0.29 −0.197
Feature: RNA
NRE
8.1e−05
1.6e−06
0.12
0.011
21015 353 21285 83 21306 62 21339 29
−0.358 −0.385 −0.358 −0.42 −0.358 −0.386 −0.358 −0.4
Feature: Replication_timing
WTS
0.24
0.51
0.59
0.36
21015 353 21285 83 21306 62 21339 29
71.313 71.823 71.313 72.587 71.324 68.928 71.313 74.865
Feature: LaminB1
NRC
0.41
0.054
0.7
0.43
21015 353 21285 83 21306 62 21339 29
1.245 1.314 1.245 1.432 1.246 1.448 1.246 1.633

**Figure S3.** Complementary to Fig. 3. **a.** Enrichment for the categorical (epi)genomic features considered when comparing the different genome-wide subsets of differentially methylated positions (DMPs) in ageing and Sotos against a control (see Methods). The y-axis represents the odds ratio (OR), the error bars show the 95% confidence interval for the OR estimate and the colour of the points codes for -$\log_{10}$(p-value) obtained after testing for enrichment using Fisher's exact test. An OR > 1 shows that the given feature is enriched in the subset of DMPs considered, whilst an OR < 1 shows that it is found less than expected. The 'Hyper-Hypo DMPs' subset results from the intersection between the hypermethylated DMPs in ageing and the hypomethylated DMPs in Sotos. The 'Hypo-Hypo DMPs' subset results from the intersection between the hypomethylated DMPs in ageing and Sotos. In grey: features that did not reach significance using a significance level of $\alpha$ = 0.01 after Bonferroni correction. **b.** Boxplots showing the distributions of scores (see Methods) for the continuous (epi)genomic features considered when comparing the different genome-wide subsets of differentially methylated positions (DMPs) in ageing and Sotos against a control (see Methods). The p-values (two-sided Wilcoxon's test, before multiple testing correction) are shown above the boxplots. The number of DMPs belonging to each subset (in green) and the median value of the feature score (in dark red) are shown below the boxplots. NFC: 'normalised fold change'; NRE: 'normalised RNA expression'; WTS: 'wavelet-transformed signals'; NRC: 'normalised read counts'. **c.** DNA methylation (beta-value) profiles for two of the clock CpG sites (cg02071305 and cg18328933). A linear model (displayed in dark grey) can be fixed to each CpG site to model the changes in beta-value with chronological age in the controls (grey). Afterwards, the difference of the Sotos samples beta-values (orange) with the controls can be estimated. **d.** Heatmap displaying the differential methylation patterns for Sotos samples (rows) when compared with controls in each one of the 353 clock CpGs (columns). Hierarchical clustering was performed in both rows and columns. RNA refers to the 'normalised RNA expression' (NRE, see Methods). H3K36me3 refers to the H3K36me3 histone modification 'normalised fold change' (NFC, see Methods). aDMPs: differentially methylated positions during ageing. EAA: epigenetic age acceleration. CCC: cell composition correction. PBMC: peripheral blood mononuclear cells. **e.** Heatmap displaying the scores for the different continuous (epi)genomic features (rows) in each one of the 353 clock CpGs (columns). The names of the features include the ENCODE ID. Hierarchical clustering was performed in both rows and columns. **f.** Same as a., but focused on the 353 clock CpG sites. **g.** Same as b., but focused on the 353 clock CpG sites.

# Figure S4

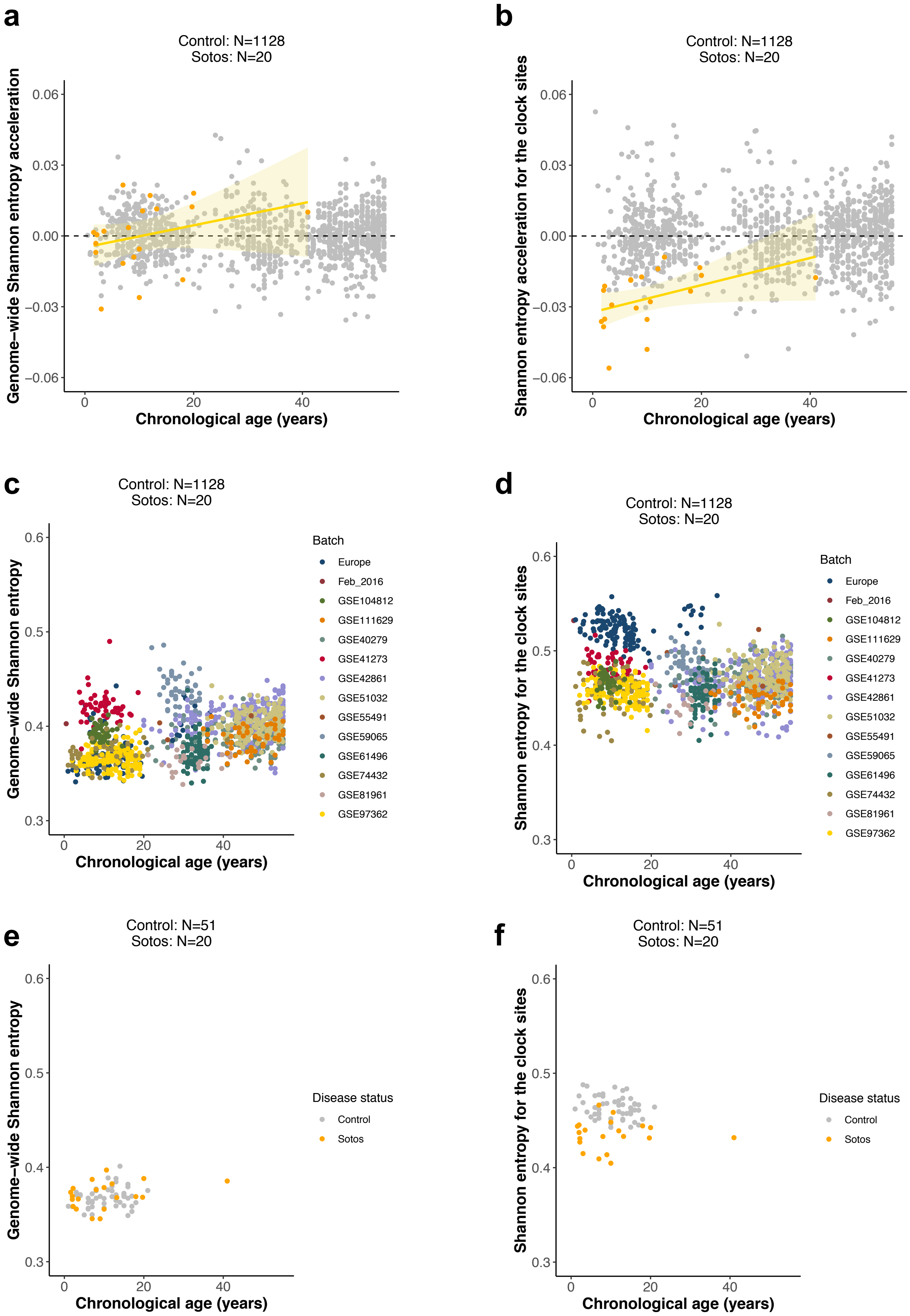
a
Control: N=1128
Sotos: N=20
Genome−wide Shannon entropy acceleration
0.06
0.03
0.00
−0.03
−0.06
0
20
40
Chronological age (years)
b
Control: N=1128
Sotos: N=20
Shannon entropy acceleration for the clock sites
0.06
0.03
0.00
−0.03
−0.06
0
20
40
Chronological age (years)
c
Control: N=1128
Sotos: N=20
Genome−wide Shannon entropy
0.6
0.5
0.4
0.3
0
20
40
Chronological age (years)
Batch
Europe
Feb_2016
GSE104812
GSE111629
GSE40279
GSE41273
GSE42861
GSE51032
GSE55491
GSE59065
GSE61496
GSE74432
GSE81961
GSE97362
d
Control: N=1128
Sotos: N=20
Shannon entropy for the clock sites
0.6
0.5
0.4
0.3
0
20
40
Chronological age (years)
Batch
Europe
Feb_2016
GSE104812
GSE111629
GSE40279
GSE41273
GSE42861
GSE51032
GSE55491
GSE59065
GSE61496
GSE74432
GSE81961
GSE97362
e
Control: N=51
Sotos: N=20
Genome−wide Shannon entropy
0.6
0.5
0.4
0.3
0
20
40
Chronological age (years)
Disease status
Control
Sotos
f
Control: N=51
Sotos: N=20
Shannon entropy for the clock sites
0.6
0.5
0.4
0.3
0
20
40
Chronological age (years)
Disease status
Control
Sotos

**Figure S4.** Complementary to Fig. 4. **a.** Scatterplot showing the relation between the genome-wide Shannon entropy acceleration (*gSEA*) and chronological age of the samples for Sotos (orange) and healthy controls (grey). Each sample is represented by one point. The yellow line represents the linear model *gSEA ~ Age*, with the standard error shown in the light yellow shade. **b.** Same as a., but using the Shannon entropy acceleration calculated only for the 353 CpG sites in the Horvath epigenetic clock (*cSEA*). **c.** Scatterplot showing the effects of the different batches on the genome-wide Shannon entropy calculations. Each sample is represented by one point and coloured according to the batch that they belong to. **d.** Same as c., but using the Shannon entropy calculated only for the 353 CpG sites in the Horvath epigenetic clock. **e.** Scatterplot showing the relation between genome-wide Shannon entropy and chronological age of the samples for Sotos (orange) and healthy controls (grey) in the GSE74432 batch (i.e. the batch where all the Sotos samples come from). Each sample is represented by one point. **f.** Same as e., but using the Shannon entropy calculated only for the 353 CpG sites in the Horvath epigenetic clock.

# Figure S5

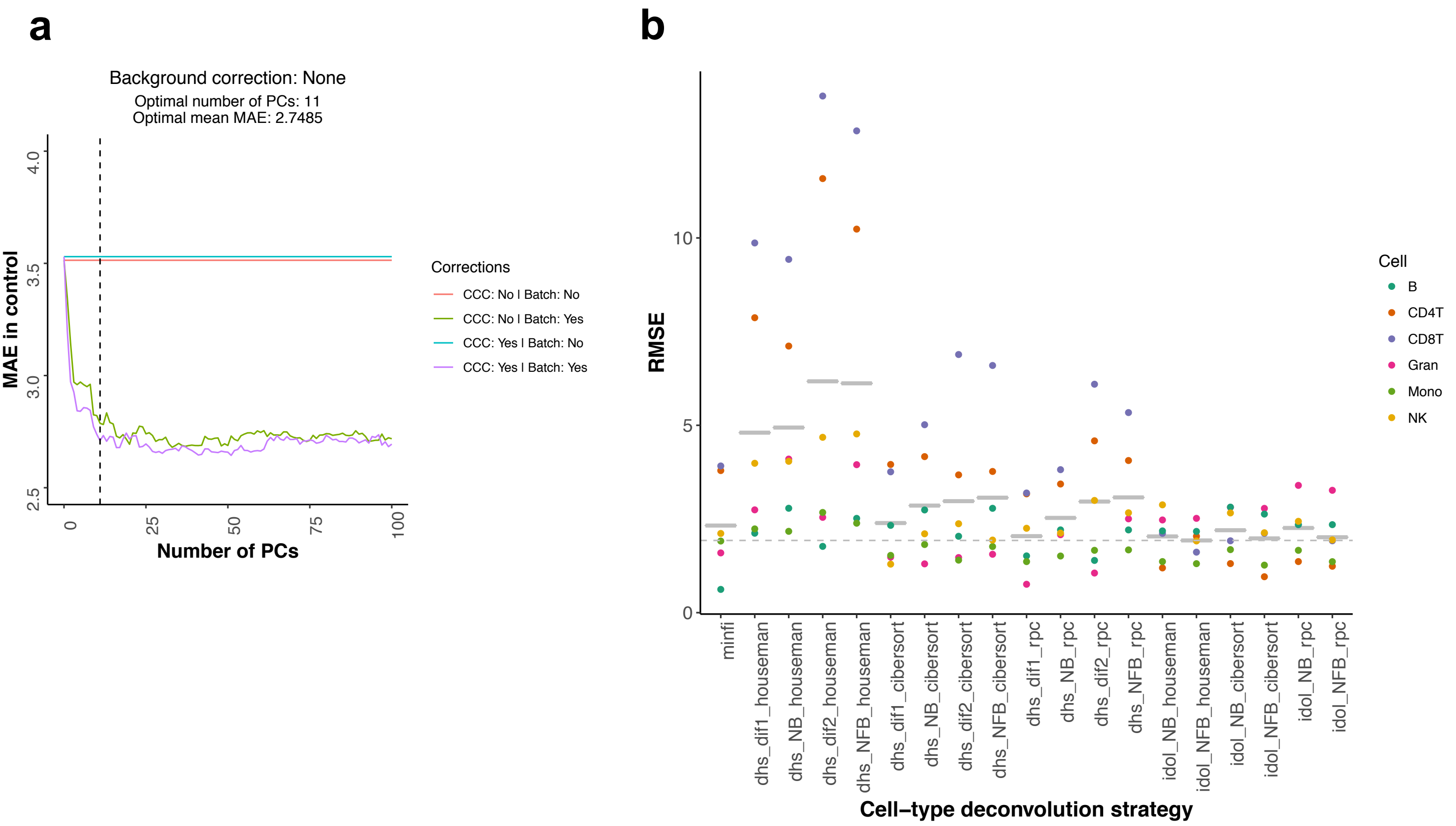

**Figure S5.** Complementary to Methods. **a.** Plot showing how the median absolute error (MAE) of the prediction in the control samples, that should tend to zero, is reduced when the PCs capturing the technical variation are included as part of the modelling strategy (see Methods). In this case, no background correction was performed, as opposed to the results in Fig. 1c. The dashed line represents the optimal number of PCs (11) that was finally used. The optimal mean MAE is calculated as the average MAE between the green and purple lines. CCC: cell composition correction. **b.** Benchmarking of the different strategies for cell-type deconvolution in blood. The x-axis shows the different strategies that were tested (a more detailed description of these strategies can be found in Additional file 4). The y-axis shows the root mean square error (RMSE) obtained when comparing our predictions with the real proportions of cells in a gold-standard dataset (GSE77797). The grey horizontal solid lines represent the mean for the RMSE across cell types and the grey dashed line the minimum of these values.
