# Supplementary figures and images for: Screening for genes that accelerate the epigenetic aging clock in humans reveals a role for the H3K36 methyltransferase NSD1
Source: Genome Biol. 2019 Aug 14;20:146. doi: 10.1186/s13059-019-1753-9 (PMC6693144; doi:10.1186/s13059-019-1753-9)

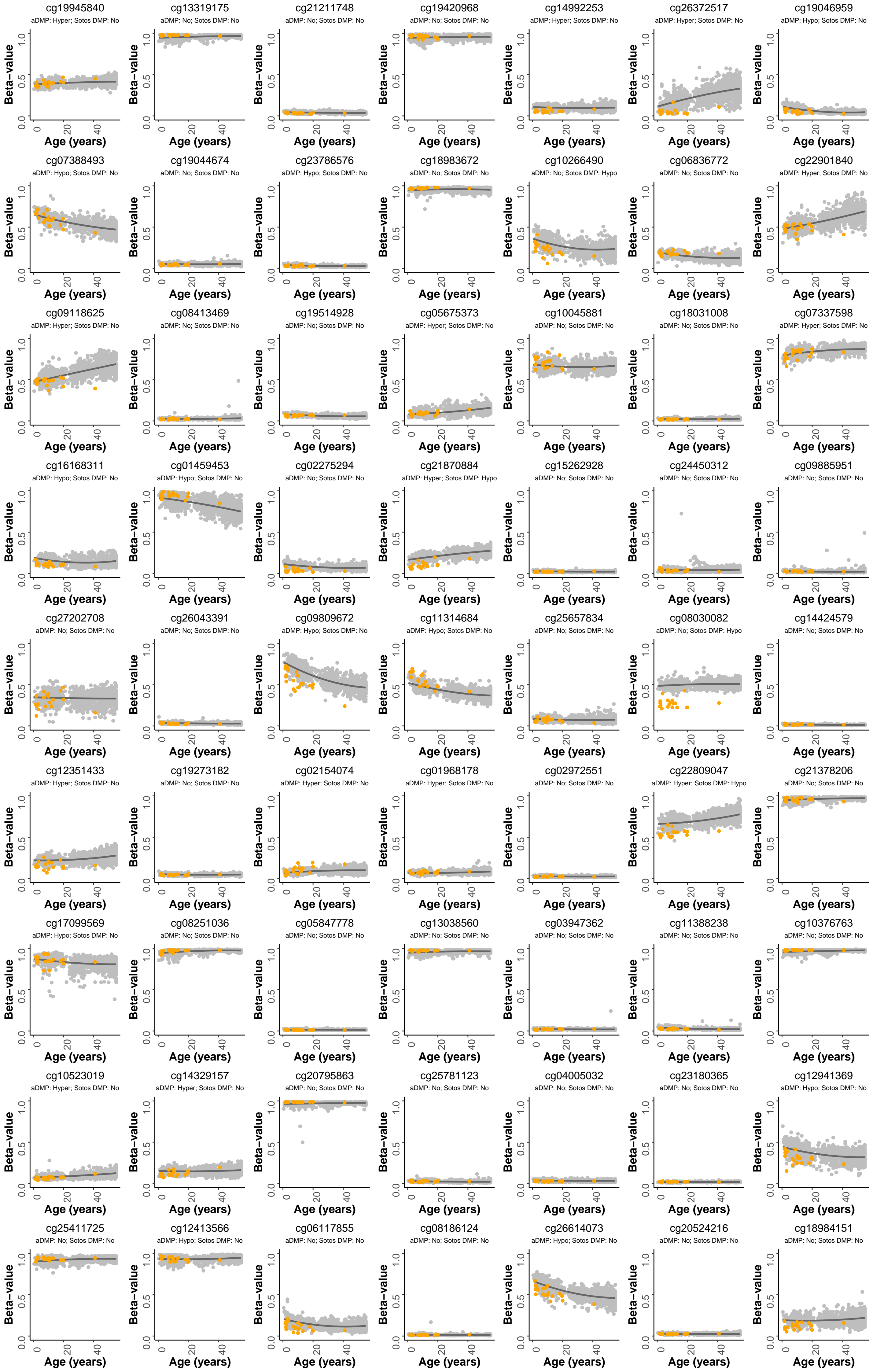

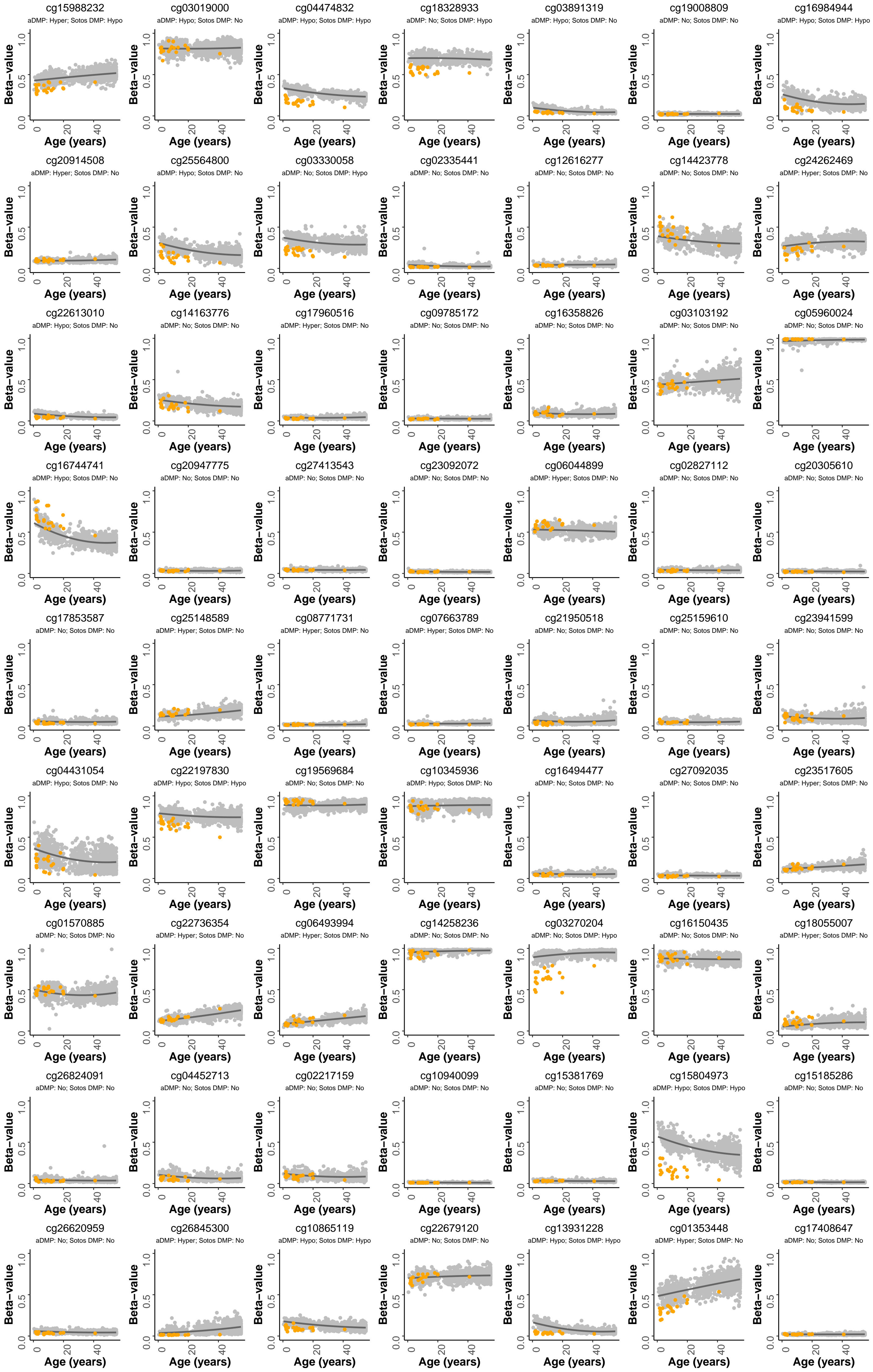

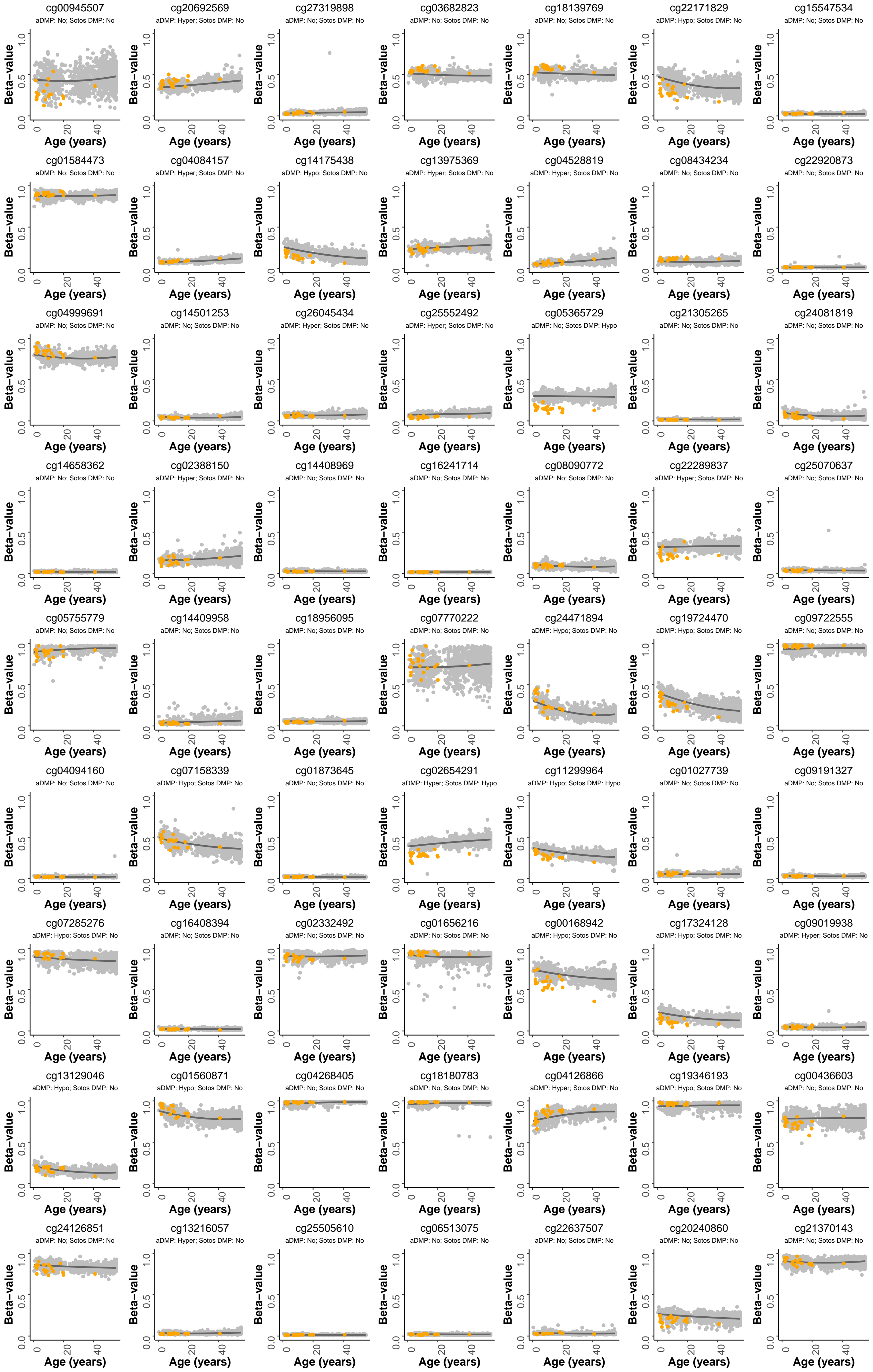

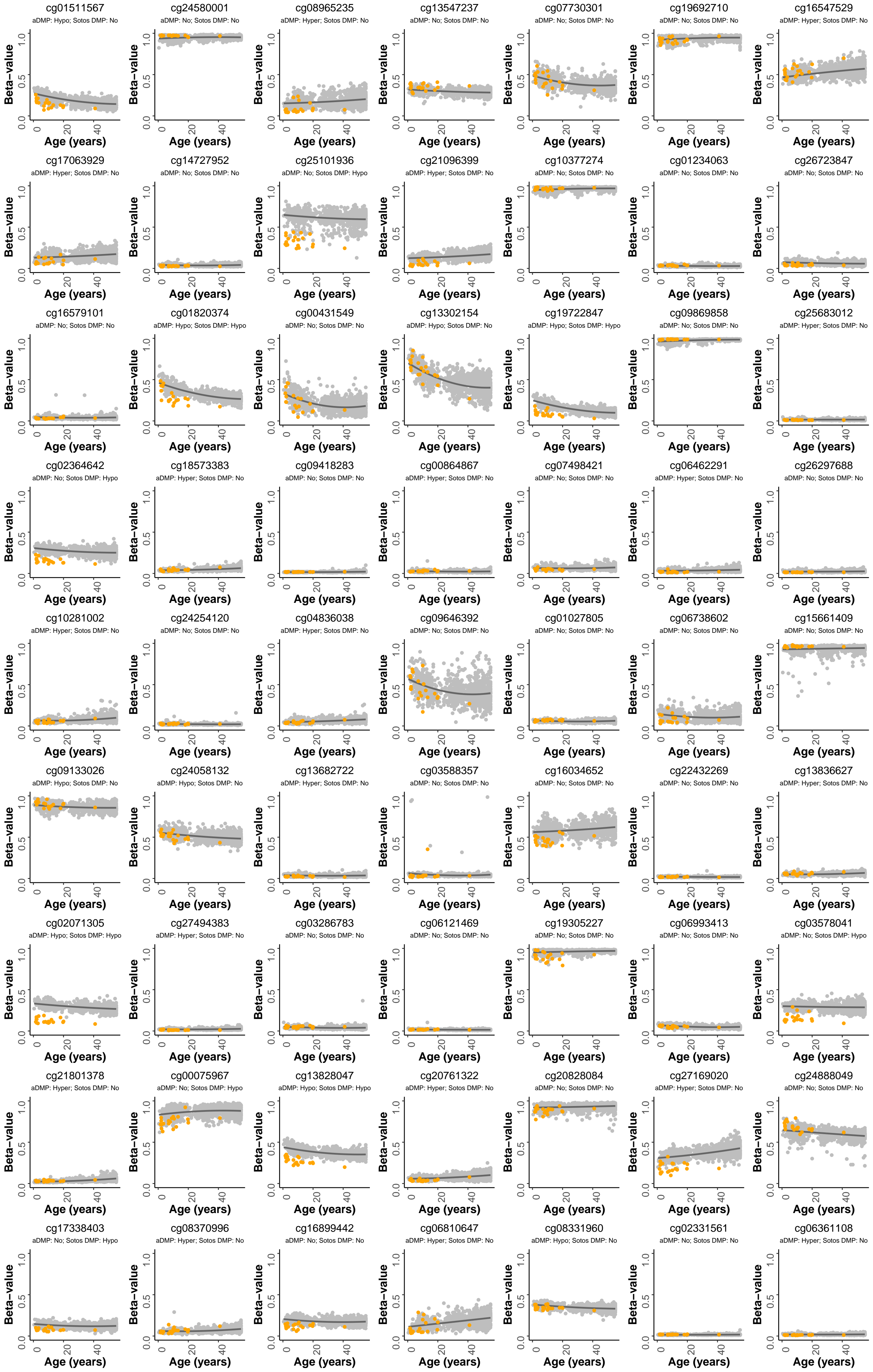

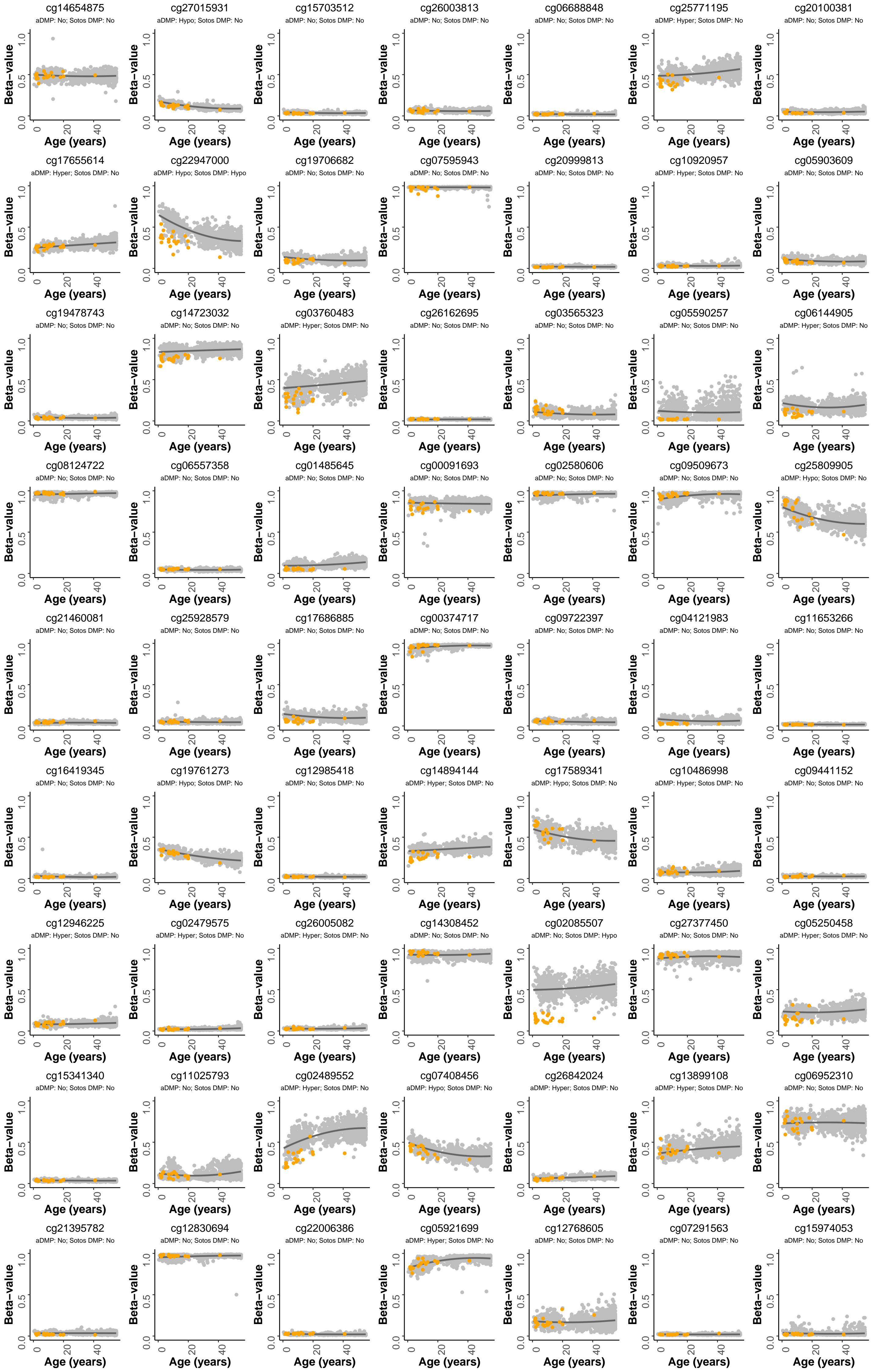

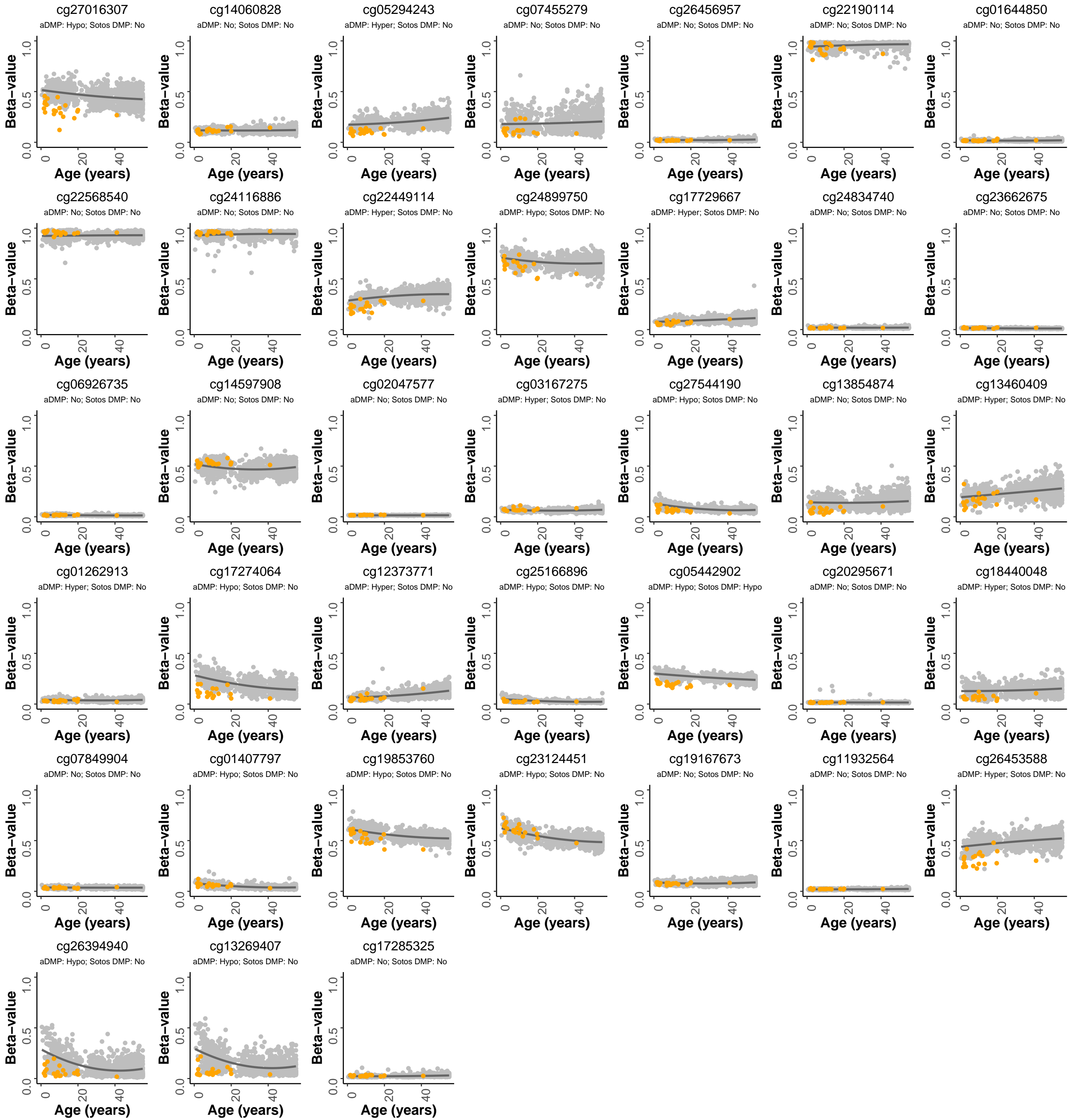

Supplement: Supplementary file 6 — DNA methylation (beta value) profiles for the 353 Horvath’s epigenetic clock CpG sites during aging for healthy individuals (gray) and Sotos patients (orange). A linear model (displayed in dark gray) can be fixed to each CpG site to model the changes in beta value with chronological age in the controls (gray). Information about whether the site is a differentially methylated position during aging (aDMP) or in Sotos patients (Sotos DMP) is also provided. Hyper, hypermethylated; Hypo, hypomethylated; No, not statistically significant after Bonferroni correction. (PDF 2811 kb) [file 13059_2019_1753_MOESM6_ESM.pdf]
