# Supplementary material for: Screening for genes that accelerate the epigenetic aging clock in humans reveals a role for the H3K36 methyltransferase NSD1
Source: Genome Biol. 2019 Aug 14;20:146. doi: 10.1186/s13059-019-1753-9 (PMC6693144; doi:10.1186/s13059-019-1753-9)
Supplement: Supplementary file 7 — Review history. (DOCX 42 kb) [file 13059_2019_1753_MOESM7_ESM.docx]

Review History

**First round of review**

**Reviewer 1**

**Are you able to assess all statistics in the manuscript, including the appropriateness of statistical tests used?** Yes, and I have assessed the statistics in my report.

**Comments to author:**

The rational of screening publically available DNA methylation profiles of patients with developmental disorders to elucidate if mutations might impact on the epigenetic clock is very interesting. The authors indicate that particularly patients with Sotos syndrome have significantly accelerated age. Sotos syndrome is in the majority of cases associated with loss-of-function heterozygous mutations in the NSD1 gene. Therefore the authors speculate that the histone H3K36 methyltransferase might be relevant for regulation of epigenetic age. The increased epigenetic age was also described few months ago in a preprint "Growth disrupting mutations in epigenetic regulatory molecules are associated with abnormalities of epigenetic aging" (bioRxiv. 2018; 477356). In this preprint the other group used the same epigenetic clock and demonstrated that also patients with Sotos syndrome with intergenic deletions revealed accelerated aging - an aspect that is not considered in this study.

I have the following specific concerns:
1) Only one epigenetic aging signature was used. To determine if age-associated changes are generally accelerated in developmental disorders the authors should compare their results with other signatures e.g. by Hannum et al. (PMID: 23177740) and Lin et al. (PMID: 26928272). Is similar EAA observed with these signatures in Sotos syndrome?
2) The authors recapitulate previous findings that Sotos syndrome patients have typical DNA methylation signature, which is particularly characterized by hypomethylation (PMID: 26690673). However, the intersections between the hypermethylated and hypomethylated DMPs in ageing and Sotos were only moderate. Furthermore, even very young Sotos syndrome patients revealed similar EAA as older patients, indicating that aging is not necessarily accelerated but there is rather a general shift to higher age (the trend in Figure 2C seems to rely particularly on one data point of a 40 year of Sotos syndrome patient). In addition, the trend in the epigenetic mitotic clock seems to rely on the same "outlier", too. These findings should at least be critically discussed. 
3) It would be interesting to understand what individual age-associated CpGs reveal accelerated epigenetic aging. This should be analyzed. The provided heatmaps are difficult to read and correlation with DNA methylation level does not provide this relevant information. 
4) It is an interesting finding that Shannon entropy is decreased in clock sites but not genome-wide of Soto patients, however, as the authors mentioned in line 367-372, this might be due to batch effects. These findings are therefore unclear, too.
5) Correlation of H3K36me3 ChIP-seq data with age-associated DNA methylation might be further elaborated (including hyper and hypomethylated regions in aging, Sotos, overlap; and different windows). For this review, the provided data does not provide clear evidence for similar histone modifications in sotos syndrome patients and aging DMPs. As it stands the message does not become very clear.
6) The authors claim a functional role for the H3K36 methyltransferase NSD1. In fact, other recent publications indicated that H3K26 trimethylation might indeed be relevant for the aging process, but this has not been tested in this study. 

Taken together, the finding of accelerated epigenetic aging in Sotos patients requires further validation - the age predictor might be falsified by the global hypomethylation associated with this disease. Furthermore, the manuscript might be strengthened by some functional data that prove the relevance for the H3K36 methyltransferase NSD1.

**Reviewer 2**

**Are you able to assess all statistics in the manuscript, including the appropriateness of statistical tests used?** Yes, and I have assessed the statistics in my report.

**Comments to author:**

This interesting and timely article contains several new/exciting results surrounding the molecular basis of epigenetic aging. I expect that the article is of broad interest to biologists because the article has many strengths: 1) exciting epigeneti clock analysis of 13 developmental disorders, 2) expert statistical analysis surrounding the removal of batch effects, 3) convincing mechanistic interpretation of the results (surrounding the DNMT3A/B and histone mark H3K36me3 e.g. page 8, lines 304-307) 4) strong discussion of results and effective literature review. 
The statistical analysis is very impressive in its own right: using control probes to minimize prediction error and to effectively get around the one limitation of the article: confounding between batch and disease status. I really liked conceptual Figure 5 and its discussion. Overall, the article greatly advances our molecular understanding of the epigenetic clock and age related DNA methylation changes. 
In my opinion, the article could be accepted "as is" but I am listing a few minor suggestions, which could be ignored.
1) I think the title of the article is sub-optimal because the phrase "screening for genes" evokes high throughput screening or GWAS. How about the following more precise title: "Epigenetic clock analysis of human developmental disorders reveals that H3K36 methyltransferase NSD1 drives epigenetic aging."
2) First sentence of abstract: replace "predict the biological age" by "predict the chronological age"
3) Results section of abstract: Personally, I think the results surrounding Shannon entropy don't need to be mentioned in the abstract. I would rather use the space to highlight other exciting findings of the article, e.g. the opposite effect in Kabuki syndrome.
4) Background or Discussion: mention a negative finding: a severe developmental disorder with multifocal developmental dysfunctions (syndrome X) was *not* associated with slow epigenetic aging: Walker et al 2015 Aging. PMID: 25991677.

5) Background: Briefly mention why you used the Horvath clock. I think it is well suited for the analysis of developmental disorders because (unlike other clocks) it is a "life course clock" that applies even to prenatal samples. Relevant citations: Horvath 2013, Horvath and Raj 2018 Nat Rev Genetics and Hoshino 2019 Scientific Reports PMID: 30842553

6) Strictly optional: Recently Horvath et al 2018 PMID: 30048243 published a new skin and blood clock which leads to very accurate age estimates in blood samples from children. I wonder whether the skin and blood clock also lends itself for detecting epigenetic age acceleration effect in Sotos syndrome ? If so, it would show that the results are highly robust. If not, no worries because the multi-tissue clock results will be of interest to the broader community.
7) Page 6, line 207 and elsewhere: I would replace "epigenetic (biological) age" by simply "epigenetic age" because "epigenetic age" is a well known and precisely defined concept. By contrast, "biological age" is a vague concept. Some readers might object to equating epigenetic age of blood with biological age. 
8) Page 7, line 265: I would replace "people" by "Individuals",
9) Page 7, lines 265-272. I wonder whether this paragraph ("Consequently,…") should be moved to the Discussion section?
10) Page 9, Discussion, lines 390 etc. Justify the claim that developmental disorders in humans represent an interesting framework for studying aging by briefly mentioning the "epigenetic clock theory of aging" from Horvath and Raj (PMID: 29643443) that postulates that epigenetic clocks are a continuous read out that connect purposeful processes in development with adverse effects later in life.

No further comments.

**Authors Response**

We thank both reviewers for their insightful comments and helpful suggestions. We are very pleased with the overall positive and supportive assessment by the reviewers who conclude that this manuscript provides novel molecular insights into the epigenetic ageing clock by making use of the DNA methylation profiles of patients with developmental disorders. We have revised our manuscript following the reviewers’ comments and suggestions and have answered all issues raised. We believe that the revised manuscript has been further strengthened and improved due to the suggestions of both reviewers and we hope that this will now be acceptable for publication.

**Point-by-point responses to the reviewers’ comments:**

**Referee 1**

Comment:

The rational of screening publicly available DNA methylation profiles of patients with developmental disorders to elucidate if mutations might impact on the epigenetic clock is very interesting. The authors indicate that particularly patients with Sotos syndrome have significantly accelerated age. Sotos syndrome is in the majority of cases associated with loss-of-function heterozygous mutations in the NSD1 gene. Therefore the authors speculate that the histone H3K36 methyltransferase might be relevant for regulation of epigenetic age. The increased epigenetic age was also described few months ago in a preprint "Growth disrupting mutations in epigenetic regulatory molecules are associated with abnormalities of epigenetic aging" (bioRxiv. 2018; 477356). In this preprint the other group used the same epigenetic clock and demonstrated that also patients with Sotos syndrome with intergenic deletions revealed accelerated aging - an aspect that is not considered in this study.

***Response:***  *We thank the reviewer for the comments and hope that our answers are satisfactory. It is worth mentioning that we acknowledge the mentioned preprint in the discussion of our manuscript (lines 512-524) and that the focus of that preprint is on de novo DNMT3A mutations in Tatton-Brown-Rahman syndrome, with our study providing deeper molecular insights into the effects of NSD1 mutations on the epigenetic ageing clock.*

I have the following specific concerns:

1) Only one epigenetic aging signature was used. To determine if age-associated changes are generally accelerated in developmental disorders the authors should compare their results with other signatures e.g. by Hannum et al. (PMID: 23177740) and Lin et al. (PMID: 26928272). Is similar EAA observed with these signatures in Sotos syndrome?

***Response:*** *We have now calculated the epigenetic ages according to three additional epigenetic clocks: Hannum’s clock, Lin’s clock and the skin-blood clock. In all cases (with the exception of the EAA_without CCC_ in the skin-blood clock) Sotos patients present accelerated epigenetic ageing when compared with the controls (see Additional file 1: Figure S2C-E and lines 284-289 and 730-739 in the main text). Therefore, these analyses clearly validate the main conclusion of this manuscript.*

2) The authors recapitulate previous findings that Sotos syndrome patients have typical DNA methylation signature, which is particularly characterized by hypomethylation (PMID: 26690673). However, the intersections between the hypermethylated and hypomethylated DMPs in ageing and Sotos were only moderate. Furthermore, even very young Sotos syndrome patients revealed similar EAA as older patients, indicating that aging is not necessarily accelerated but there is rather a general shift to higher age (the trend in Figure 2C seems to rely particularly on one data point of a 40 year of Sotos syndrome patient). In addition, the trend in the epigenetic mitotic clock seems to rely on the same "outlier", too. These findings should at least be critically discussed.

***Response****: The reviewer is absolutely right when pointing out that the DMPs intersections are only moderate, which we also highlight in lines 333-334 of the main text. This could be caused by the stringent criteria used to select the DMPs (Bonferroni correction instead of FDR for example). Furthermore, it is important to mention that we do not claim that all the ageing DMPs are related to NSD1 function, but rather that the subset that overlaps is very likely related with H3K36 methylation maintenance. Naturally, there will be many more processes that contribute to the changes observed in the epigenome with age, as mentioned in lines 380-386 of the main text. Given the current sample size of Sotos patients, it is difficult to be certain of whether the DNA methylation changes observed accumulate progressively, are present since birth, or a combination of both. The reviewer also makes an important comment regarding the effects of the 41-year-old Sotos patient on the conclusions regarding EAA and pcgtAge. We have now critically discussed the effects of removing this patient from the analyses (lines 272-282 and 299-301 in the main text).*

3) It would be interesting to understand what individual age-associated CpGs reveal accelerated epigenetic aging. This should be analyzed. The provided heatmaps are difficult to read and correlation with DNA methylation level does not provide this relevant information.

***Response****: We have now included individual scatterplots for the Horvath sites, which also contain information on whether the site is an aDMP in the controls and whether the site is differentially methylated in Sotos patients. (see Additional File 6). This way heatmaps are clarified and it is possible to clearly identify those sites that display accelerated epigenetic ageing.*

4) It is an interesting finding that Shannon entropy is decreased in clock sites but not genome-wide of Soto patients, however, as the authors mentioned in line 367-372, this might be due to batch effects. These findings are therefore unclear, too.

***Response****: The reviewer raises an important concern regarding the validity of the entropy analyses when comparing Sotos syndrome patients against all the controls. We have now made the same comparisons but considered only the control samples that are part of the same batch as the Sotos samples (GSE74432), therefore avoiding any potential confounding due to batch effect. The findings are still valid after this analysis (Additional file 1: Figure S4E-F, see lines 404-407 and 424-427 in main text).*

5) Correlation of H3K36me3 ChIP-seq data with age-associated DNA methylation might be further elaborated (including hyper and hypomethylated regions in aging, Sotos, overlap; and different windows). For this review, the provided data does not provide clear evidence for similar histone modifications in Sotos syndrome patients and aging DMPs. As it stands the message does not become very clear.

***Response****: The information requested by the reviewer is already provided in the supplementary data of the manuscript (Additional File 1: Figure S3B). We have made a more explicit reference in the main text (lines 354-355). Furthermore, the median normalised fold change (NFC) follows the same direction when comparing against the control in 6/7 of the histone marks assessed (Additional File 1: Figure S3B), both when comparing the ‘Hyper’ aDMPs with ‘Hyper’ Sotos DMPs (only H3K4me3 differs) and the ‘Hypo’ aDMPs with ‘Hypo’ Sotos DMPs (only H3K9me3 differs). Therefore, we believe that, overall, this is enough unbiased evidence of a shared genomic context between ageing and Sotos DMPs.*

6) The authors claim a functional role for the H3K36 methyltransferase NSD1. In fact, other recent publications indicated that H3K36 trimethylation might indeed be relevant for the aging process, but this has not been tested in this study.

***Response****: A functional role for the H3K36 methylation machinery during human ageing is suggested in this study (Figure 5). This is indeed supported by evidence in other model organisms. We have made clearer that further experiments are required to validate this hypothesis in mammals (in humans: lines 598-599; in the mouse, see lines 602-604). This goes beyond the scope of this manuscript and would require another study in itself.*

Taken together, the finding of accelerated epigenetic aging in Sotos patients requires further validation - the age predictor might be falsified by the global hypomethylation associated with this disease. Furthermore, the manuscript might be strengthened by some functional data that prove the relevance for the H3K36 methyltransferase NSD1.

***Response****: We have robustly shown that accelerated epigenetic ageing in Sotos patients is observed using four different types of epigenetic clocks, which use different subsets of CpG sites and were trained on different datasets and tissues.*

*The falsification by global hypomethylation in Sotos patients is indeed an important concern. We have simulated different levels of global hypomethylation in our healthy control patients:*

a. *‘Sotos-like’ levels of hypomethylation. There are 15062 significant hypomethylated DMPs in our Sotos patients (3.52% of the total number of probes in the array). These Sotos DMPs have a median effect size of -0.18 on the beta-values. Therefore, we have randomly selected ~3.52% of the CpG sites in the Horvath epigenetic clock (12/353) and reduced their beta-values by 0.18 in our population of healthy controls. The median EAA for the population was then calculated and this process repeated 1000 times (to generate a distribution).*
b. *Extreme hypomethylation. In this case 50% of the CpG sites of the Horvath clock (176/353) were reduced their beta-values by 0.5 in a procedure similar to the one described in a).*

*Finally, we have also plotted the median EAA actually observed in our Sotos patients (in orange, no distribution in this case since we only have one population observed). The results can be visualised in the following plot:*

*Under ‘Sotos-like’ levels of global hypomethylation, the EAA is barely affected (EAA~0 years). Under extreme levels of global hypomethylation the median EAA is ~ 6 years. For our Sotos patients, the median EAA is 7.64 years. Therefore, it is highly unlikely that global hypomethylation explains the EAA observed in our Sotos patients.*

*Given that these responses will be published, this part of the analysis will not be included in the main manuscript in order to ease the reading flow.*

**Referee 2**

This interesting and timely article contains several new/exciting results surrounding the molecular basis of epigenetic aging. I expect that the article is of broad interest to biologists because the article has many strengths: 1) exciting epigenetic clock analysis of 13 developmental disorders, 2) expert statistical analysis surrounding the removal of batch effects, 3) convincing mechanistic interpretation of the results (surrounding the DNMT3A/B and histone mark H3K36me3 e.g. page 8, lines 304-307) 4) strong discussion of results and effective literature review.

The statistical analysis is very impressive in its own right: using control probes to minimize prediction error and to effectively get around the one limitation of the article: confounding between batch and disease status. I really liked conceptual Figure 5 and its discussion. Overall, the article greatly advances our molecular understanding of the epigenetic clock and age-related DNA methylation changes.

In my opinion, the article could be accepted "as is" but I am listing a few minor suggestions, which could be ignored.

***Response:*** *We thank the reviewer for the positive and encouraging comments and hope that our answers are satisfactory.*

1) I think the title of the article is sub-optimal because the phrase "screening for genes" evokes high throughput screening or GWAS. How about the following more precise title: "Epigenetic clock analysis of human developmental disorders reveals that H3K36 methyltransferase NSD1 drives epigenetic aging."

***Response:*** *We thank the reviewer for the updated title suggestion. We have discussed the suggestion and we believe that further evidence is required to be able to claim that NSD1 ‘drives’ epigenetic ageing. Therefore, we will leave the title as it currently stands.*

2) First sentence of abstract: replace "predict the biological age" by "predict the chronological age".

***Response:*** *Even though epigenetic clocks are indeed trained on chronological age, they have been shown (at least Horvath’s clock) to capture a version of biological age (as determined by an epigenetic biomarker). Therefore, we will leave the abstract as it currently stands.*

3) Results section of abstract: Personally, I think the results surrounding Shannon entropy don't need to be mentioned in the abstract. I would rather use the space to highlight other exciting findings of the article, e.g. the opposite effect in Kabuki syndrome.

***Response:*** *We believe further validation is required to be certain about the negative epigenetic age acceleration observed in Kabuki syndrome and including this observation in the abstract could lead to confusion if the reader does not read the entire manuscript. On the contrary, the fact that the CpG sites that constitute the epigenetic clock have different Shannon entropy properties when compared with the average sites in the genome is probably more relevant from a biological point of view. Therefore, we will leave the abstract as it currently stands.*

4) Background or Discussion: mention a negative finding: a severe developmental disorder with multifocal developmental dysfunctions (syndrome X) was ***not*** associated with slow epigenetic aging: Walker et al 2015 Aging. PMID: 25991677.

***Response:*** *This is a great suggestion and we have included it in the main text (lines 129-131).*

5) Background: Briefly mention why you used the Horvath clock. I think it is well suited for the analysis of developmental disorders because (unlike other clocks) it is a "life course clock" that applies even to prenatal samples. Relevant citations: Horvath 2013, Horvath and Raj 2018 Nat Rev Genetics and Hoshino 2019 Scientific Reports PMID: 30842553

***Response:*** *This is a great suggestion and we have included it in the main text (lines 167-169).*

6) Strictly optional: Recently Horvath et al 2018 PMID: 30048243 published a new skin and blood clock which leads to very accurate age estimates in blood samples from children. I wonder whether the skin and blood clock also lends itself for detecting epigenetic age acceleration effect in Sotos syndrome ? If so, it would show that the results are highly robust. If not, no worries because the multi-tissue clock results will be of interest to the broader community.

***Response:*** *As previously mentioned, we have now validated the EAA observed in Sotos patients using, besides Horvath’s clock, other three epigenetic clocks, including the skin-blood clock (see Additional file 1: Figure S2C-E and lines 284-289 and 730-739 in the main text).*

7) Page 6, line 207 and elsewhere: I would replace "epigenetic (biological) age" by simply "epigenetic age" because "epigenetic age" is a well known and precisely defined concept. By contrast, "biological age" is a vague concept. Some readers might object to equating epigenetic age of blood with biological age.

***Response:*** *We agree that biological age is a vaguer and biomarker-specific concept. Nevertheless, for some readers, it may still be useful to equate biological age with epigenetic age in the context of the epigenetic ageing clock. As such, we have left this untouched.*

8) Page 7, line 265: I would replace "people" by "Individuals"

***Response:*** *This is now corrected in the main text.*

9) Page 7, lines 265-272. I wonder whether this paragraph ("Consequently,…") should be moved to the Discussion section?

*Response: We find useful to include this paragraph at the end of the results section in order to summarise the main findings of this part of the analysis.*

10) Page 9, Discussion, lines 390 etc. Justify the claim that developmental disorders in humans represent an interesting framework for studying aging by briefly mentioning the "epigenetic clock theory of aging" from Horvath and Raj (PMID: 29643443) that postulates that epigenetic clocks are a continuous read out that connect purposeful processes in development with adverse effects later in life.

***Response:*** *This is a great suggestion and we have included it in the main text (lines 442-444).*

No further comments.

**Second round of review**

**Reviewer 1**

I want to thank the authors for addressing each of my comments. I still feel that more effort should have been taken to demonstrate the functional role for the H3K36 methyltransferase NSD1, but I understand that this might be beyond this study.

**Reviewer 2**

Thank you for carefully addressing all of my comments.
No further comments.
